# Subcutaneous administration of a novel TRPM8 antagonist reverses cold hypersensitivity while attenuating the drop in core body temperature

**DOI:** 10.1111/bph.16429

**Published:** 2024-05-24

**Authors:** Michael S. Gold, Jorge B. Pineda-Farias, David Close, Smith Patel, Paul A. Johnston, Sean D. Stocker, V. Blair Journigan

**Affiliations:** 1 Department of Neurobiology, Pittsburgh Center for Pain Research, University of Pittsburgh, Pittsburgh, Pennsylvania, USA; 2 Department of Pharmaceutical Sciences, School of Pharmacy, University of Pittsburgh, Pittsburgh, Pennsylvania, USA; 3 Computational Chemical Genomics Screening Center, School of Pharmacy, University of Pittsburgh, Pittsburgh, Pennsylvania, USA

**Keywords:** cancer, chemotherapy, neuropathic pain, PBMC, thermoregulation

## Abstract

**Background and Purpose:**

We extend the characterization of the TRPM8 antagonist VBJ103 with tests of selectivity, specificity and distribution, therapeutic efficacy of systemic administration against oxaliplatin-induced cold hyperalgesia and the impact of systemic administration on core body temperature (CBT).

**Experimental Approach:**

Selectivity at human TRPA1 and TRPV1 as well as in vitro safety profiling was determined. Effects of systemic administration of VBJ103 were evaluated in a model of oxaliplatin-induced cold hyperalgesia. Both peripheral and centrally mediated effects of VBJ103 on CBT were assessed with radiotelemetry.

**Key Results:**

VBJ103 had no antagonist activity at TRPV1 and TRPA1, but low potency TRPA1 activation. The only safety liability detected was partial inhibition of the dopamine transporter (DAT). VBJ103 delivered subcutaneously dose-dependently attenuated cold hypersensitivity in oxaliplatin-treated mice at 3, 10 and 30 mg·kg^−1^ (n = 7, *P* < 0.05). VBJ103 (30 mg·kg^−1^) antinociception was influenced by neither the TRPA1 antagonist HC-030031 nor the DAT antagonist GBR12909. Subcutaneous administration of VBJ103 (3, 10 and 30 mg·kg^−1^, but not 100 or 300 mg·kg^−1^, n = 7) decreased CBT (2°C). Intraperitoneal (i.p.) administration of VBJ103 (3, 10 and 30 mg·kg^−1^) dose-dependently decreased CBT to an extent larger than that detected with subcutaneous administration. Intracerebroventricular (i.c.v.) administration (306 nmol/1 μL; n = 5) did not alter CBT.

**Conclusions and Implications:**

We achieve therapeutic efficacy with subcutaneous administration of a novel TRPM8 antagonist that attenuates deleterious influences on CBT, a side effect that has largely prevented the translation of TRPM8 as a target.

## 1 | INTRODUCTION

The weight of available evidence supports two distinct mechanisms as primarily responsible for cold hypersensitivity observed following nerve injury in general, and chemotherapy-induced peripheral neuropathy in particular. These include the transient receptor potential (TRP) melastatin 8 ion channel (TRPM8) and the ankyrin 1 subtype, TRPA1. TRPM8 knockout mice are not only unresponsive to drops in environmental temperature (Bautista et al., 2007; Colburn et al., 2007; Dhaka et al., 2007) but demonstrate little, if any, detectable increases in sensitivity to cold stimuli following traumatic nerve injury (Colburn et al., 2007). Comparable results have been reported with TRPM8 antagonists, at least with respect to nerve injury-induced cold hypersensitivity (Calvo et al., 2012; Chaudhari et al., 2013; De Caro et al., 2019, 2018; Parks et al., 2011). In contrast, cold hypersensitivity induced by the chemotherapeutic oxaliplatin was significantly attenuated in the TRPA1-knock out mice (Nassini et al., 2011). Furthermore, direct inhibition of TRPA1 with the selective antagonist HC-030031 suppresses cold hypersensitivity in oxaliplatin-treated mice (Trevisan, Materazzi, et al., 2013; Zhao et al., 2012).

While there is compelling evidence in support of both TRPM8 and TRPA1 as underlying mechanisms of cold hypersensitivity, we have pursued TRPM8 as a therapeutic target based on our promising data with local administration of our novel selective TRPM8 antagonist (Journigan et al., 2020). Furthermore, there is evidence from reciprocal point mutation studies involving 13-lined ground squirrels, obligate hibernators able to drop core body temperature (CBT) (Cooper et al., 2016; Junkins et al., 2022; Mohr et al., 2020), and non-hibernating mammals, suggesting that residues on TRPM8 underlying channel activation in response to drops in CBT (Matos-Cruz et al., 2017) are spatially distinct from those responsible for the detection of environmental cold stimuli (Kuhn et al., 2013; Voets et al., 2007; Winking et al., 2012). If so, we predicted that it should be possible to block TRPM8 channels contributing to cold hypersensitivity following nerve injury while avoiding the impact of TRPM8 antagonists on CBT. This side effect has served as one of the primary barriers to the translation of TRPM8 antagonists (Knowlton et al., 2011; Patel et al., 2014).

Consistent with our prediction, we were able to demonstrate attenuation of oxaliplatin-induced cold hypersensitivity while mitigating the drop in CBT. However, this effect appeared to be due to the route of administration rather than a differential activation/inhibition of TRPM8. That is, the drop in CBT to VBJ103 was attenuated by subcutaneous versus intraperitoneal administration. Furthermore, we demonstrate that neither TRPA1 nor DAT inhibition contributes to the antinociceptive effect of VBJ103.

## 2 | METHODS

### 2.1 | *N*-(Spiro[4.5]decan-8-yl)-[1,1′-biphenyl]-4-carboxamide (VBJ103)

VBJ103 was resynthesized according to Journigan et al. (2020) and optimized using the following procedure: To a stirred solution of [1,1′-biphenyl]-4-carboxylic acid (12.2 g, 61.7 mmol, 1.8 equiv) in tetrahydrofuran (THF) (343 ml, 0.1 M) was added N-ethyl-N′-(3-dimethylaminopropyl)carbodiimide hydrochloride (EDCI) (12.3 g, 64.1 mmol, 1.87 equiv), 1-hydroxybenzotriazole hydrate (HOBt) (9.82 g, 64.1 mmol, 1.87 equiv) and Et_3_N (18.9 ml, 135.6 mmol, 3.96 equiv), and the reaction was stirred at room temperature for 1 h. To this mixture was added a solution of spiro[4.5]decan-8-amine hydrochloride (6.5 g, 34.2 mmol, 1.0 equiv), and the reaction was stirred at 40°C for 22 h. The reaction was diluted with EtOAc and H_2_O. The combined organic layers were dried over Na_2_SO_4_, filtered and concentrated. The reaction was repeated on a 1.23 g scale, and the crude material was combined with above. The residue was purified 2× by automated flash chromatography using dichloromethane/diethyl ether (1/99 → 9/91) to afford 9.17 g of the title material in 67% yield. ^1^H NMR and mass spec are consistent with our previously reported values (Journigan et al., 2020). Anal. calcd for C_23_H_27_NO: C, 82.84; H, 8.16; N, 4.20. Found: C, 82.88; H, 8.16; N, 4.21. VBJ103 was recrystallized from dioxane to provide clear colourless, blade-like crystals.

### 2.2 | Crystallization and X-ray diffraction experiments of VBJ103

A clear colourless, blade-like specimen of C_25_H_31_NO_2_, approximate dimensions 0.030 mm × 0.060 mm × 0.180 mm, was used for the X-ray crystallographic analysis. The X-ray intensity data were measured on a Bruker X8 Prospector Ultra IMuS (CuKα, λ = 1.54178 Å) system equipped with a Bruker Smart Apex II CCD detector.

The total exposure time was 9.87 h. The integration of the data using a triclinic unit cell yielded a total of 45,097 reflections to a maximum θ angle of 68.30° (0.83 Å resolution), of which 7428 were independent (average redundancy 6.071, completeness = 97.5%, R_int_ = 3.27%, R_sig_ = 2.58%) and 6749 (90.86%) were greater than 2σ(F^2^). The final cell constants of a = 9.7935(3) Å, b = 10.0923(3) Å, c = 24.2140(7) Å, α = 80.120(2)°, β = 84.886(2)°, γ = 61.7040(10)°, volume = 2075.98(11) Å^3^, are based upon the refinement of the XYZ-centroids of 9763 reflections above 20 σ(I) with 7.412° < 2θ < 136.5°. Data were corrected for absorption effects using the Multi-Scan method (TWINABS). The ratio of minimum to maximum apparent transmission was 0.857. The calculated minimum and maximum transmission coefficients (based on crystal size) are 0.6500 and 0.7500.

The structure was solved and refined using the Bruker SHELXTL Software Package, using the space group P-1, with Z = 4 for the formula unit, C_25_H_31_NO_2_. The final anisotropic full-matrix least-squares refinement on F^2^ with 506 variables converged at R1 = 3.21%, for the observed data and wR2 = 9.01% for all data. The goodness-of-fit was 1.008. The largest peak in the final difference electron density synthesis was 0.138 e^−^/Å^3^, and the largest hole was −0.183 e^−^/Å^3^ with an RMS deviation of 0.038 e^−^/Å^3^. On the basis of the final model, the calculated density was 1.208 g·cm^−3^ and F(000), 816 e^−^.

### 2.3 | (*S*)-1-Phenylethyl(2-aminoethyl) (4-(benzyloxy)-3-methoxybenzyl)carbamate hydrochloride (PBMC· HCl)

PBMC (catalogue # 10–1413) was synthesized by Focus Biomolecules, Plymouth Meeting PA, USA, using (*S*)-(−)-1-phenylethanol (Lampe et al., 2007). PBMC was converted the HCl salt via addition of HCl/dichloromethane (DCM) and used for the telemetry studies.

### 2.4 | Chemicals

Capsaicin, ruthenium red and GBR 12909·2HCl were obtained from Tocris (Minneapolis, USA). Capsazepine, allyl isothiocyanate (AITC) and methyl cellulose (Methocel^®^ A15C, medium viscosity, 1200–1800 mPa.s) were purchased from Millipore Sigma (Burlington, USA). A-967079, HC-030031 and 2-hydroxypropyl-β-cyclodextrin (HP-β-CD) were purchased from Cayman Chemical (Ann Arbor, USA). PBMC·HCl was obtained from Focus Biomolecules (Plymouth Meeting, USA) (custom synthesis). Galanin-like peptide was obtained from Bachem (Torrance, USA), and oxaliplatin was obtained from Fisher Scientific (Hampton, USA). Kolliphor HS 15 was obtained from Oakwood Chemical (Estill, USA).

For the dose–response studies of VBJ103 in the oxaliplatin model as well as determination of brain levels, VBJ103 was suspended in a vehicle solution of 1% DMSO/4% Cremophor EL/95% sterile saline (bacteriostatic 0.9% sodium chloride, Hospira (Lake Forest, USA). To assess any contributing role of TRPA1, both HC-030031 and VBJ103 were suspended in 0.5% methyl cellulose in water. AITC was dissolved in the aforementioned vehicle. To assess any contributing role of the dopamine transporter, both GBR12909 and VBJ103 were administered in 1:1 DMSO: water. For the telemetry studies using s.c. or i.p. administration, PBMC was dissolved in a vehicle solution of 10% Kolliphor HS 15/20% PEG-200 in sterile saline as described by Knowlton et al. (2011), as was GBR12909. VBJ103 (100 or 300 mg·kg^−1^, s.c.) was suspended in this vehicle solution. For the telemetry studies with i.c.v. administration, galanin-like peptide was dissolved in saline. VBJ103 was dissolved in 94% DMSO/6% 2-hydroxypropyl-β-cyclodextrin.

### 2.5 | Heterologous TRP channel expression and calcium influx fluorescence detection

The human embryonic kidney cell line (HEK293T, CRL-1573, RRID: CVCL_0045) was purchased from the American Type Culture Collection (Manassas, USA) and was maintained in Dulbecco’s Minimal Essential Medium (DMEM, Cellgro10013CV, Corning, Tewksbury, USA) that was supplemented with 10% fetal bovine serum (Gemini Bio-products), 2-mM L-glutamine (Invitrogen) and 100 U/mL penicillin and streptomycin (Invitrogen) and cultured in an incubator at 37°C, 5% CO_2_ and 95% humidity. Human TRPV1 and TRPA1 plasmid DNA (gift from D. Julius) were transiently bulk transfected individually into HEK293T cells using the FuGene HD transfection reagent (TFR, Promega, Madison, WI, USA) at a 3:1 ratio (TFR:DNA). Transfected cells were seeded at 10,000 cells per well in a volume of 25 μl into black walled collagen coated 384-well microtiter plates and cultured in an incubator at 37°C, 5% CO_2_ and 95% humidity for 48 h prior to the initiation of calcium mobilization experiments performed on a FlexStation 3 (Molecular Devices, Sunnyvale, CA, USA) platform equipped with a 16-channel robotic pipettor head and FLIPR tetra pipette tips. At 46 h post transfection, cell monolayers were preloaded with 25 μl of the FLIPR Calcium 6 dye (Molecular Devices) for 2 h in an incubator at 37°C, 5% CO_2_ and 95% humidity according to the manufacturer’s instructions. The plate was transferred to a FlexStation 3 microplate reader. Relative fluorescence units (RFUs) (Ex. 485 nm, Em 525 nm) were then acquired at 37°C in the kinetic mode. The run time for the agonist (1st) and antagonist (2nd) reads were 60 s each, for a total run time of 120 s, with an interval of 3.1 s between reads in the same well to allow all 16 wells in a column to be read sequentially, and the photomultiplier tube (PMT) level was set at medium, with 6 flashes per read. Baseline Fluo-6 RFUs were acquired for 18 s prior to the addition of compound in both the agonist and antagonist reads. For the first addition agonist read, the volume in the well was 50 μl, the pipette height was set at 40 μl, and 12.5 μl of the test compound was delivered at a speed of 2 μl·s^−1^ after the baseline RFU’s acquisition period at 19 s, and the RFUs were acquired for an additional 41 s. For the second addition antagonist read, the volume in the well was 62.5 μl, the pipette height was set at 40 μl, and 12.5 μl of an EC_80_ agonist concentration was delivered at a speed of 2 μl/s after the baseline RFU’s acquisition period at 19 s, and the RFUs were acquired for an additional 41 s. The FlexStation 3 instrument settings, automated pipetting and data collection were all controlled by the SoftMax Pro software, version 7.1.2.

### 2.6 | FlexStation-3 data processing and analysis

To analyse the agonist and antagonist Ca^2+^ mobilization Fluo-6 RFU’s kinetic data, the RFU’s readings acquired throughout the 60-s acquisition period for each individual well were divided by the average RFU values acquired during the first 18-s baseline acquisition period from the same well to calculate a fold over baseline (FOB) ratio. The FOB ratio data (Y axis) was plotted against time (X axis), and GraphPad Prism 10.1 software was used to calculate the area under the curve (AUC) for the 19 to 60 s acquisition period. To derive agonist effective concentration 50% (EC_50_) values, we used GraphPad Prism 10.1 software to plot and fit the FOB-AUC data versus the log_10_ compound concentration using the four parameter logistical model equation Y = Bottom + [Top − Bottom]/[1 + 10^(LogEC_50_ − X) − HillSlope]. To derive antagonist inhibitory concentration 50% (IC_50_) values against EC_80_ agonist concentrations, we used GraphPad Prism 10.1 software to plot and fit the FOB-AUC data versus the log_10_ compound concentration using the variable slope model equation Y = Bottom + (Top − Bottom)/(1 + 10^([LogIC_50_ − X]*HillSlope)). Representative FOB-AUC curves from one of two to three independent experiments that were conducted in 11- to 15-point concentration response assays performed in triplicate (n = 3) wells for each compound concentration are presented. Symbols and error bars represent the mean ± sd (n = 3) FOB-AUC values at each compound concentration.

### 2.7 | Safety pharmacology studies

Assessment of the safety profile of VBJ103 across 78 assays using recombinant human targets was performed with the SAFETYscan E/IC50 ELECT service by Eurofins DiscoverX (San Diego, CA, USA). Data represent a single experiment and therefore should be considered preliminary. The assays were performed utilizing PathHunter enzyme fragment complementation (EFC) technology, FLIPR^®^-based cellular screening assays, KINOME*scan* kinase binding assays and a variety of enzymatic assays. Further potential functional activity at opioid receptors was performed in the gpcrSCAN service by Eurofins DiscoverX. Hit Hunter^®^ cAMP assays were carried out using Enzyme Fragment Complementation (EFC) with β-galactosidase (β-Gal) as the functional reporter.

### 2.8 | Positive allosteric modulator (PAM) screen at μ, δ and κ opioid receptors

Assessment of potential positive allosteric modulator (PAM) activity at μ, δ and κ opioid receptors was performed with the DiscoverX HitHunter cAMP XS+ assay by Eurofins DiscoverX (San Diego, CA, USA).

### 2.9 | Determination of partition coefficient (logD)

The partition coefficient (logD) at pH 7.4 of VBJ103 was determined using the shake flask method by Absorption Systems (Exton, PA, USA). The buffer was prepared by combining 50 ml of 0.2-M solution of KH_2_PO_4_ with 150 ml of dH_2_O and then adjusting to pH 7.4 with 10-N NaOH. In a single incubation, 15 μl of a 10-mM DMSO solution of each test article was added to test tubes which contained 0.75 ml of n-octanol and 0.75 ml of pH 7.4 phosphate buffer. Testosterone was also introduced to each tube as an internal control, also at a dosing concentration of 100 μM. These samples were gently mixed on a benchtop rotator for 1 h at room temperature. The tubes were then removed from the rotator, and the aqueous and organic phases were allowed to separate for 1 h. An aliquot of the organic layer was taken and diluted 200-fold into a mixture of 1:1 buffer: acetonitrile (ACN). An aliquot of the aqueous layer was taken and diluted 2-fold, 10-fold and 200-fold into a mixture of 1:1 buffer: ACN. All samples were assayed by LC–MS/MS on a Thermo BDS Hypersil C18 column (30 × 2.1 mm, 3 μm) using electrospray ionization, as previously reported (Journigan et al., 2020).

### 2.10 | Animal experimental design

Studies were designed to generate groups of equal size. In two of the oxaliplatin experiments, mice died during the administration of the chemotherapy, and these mice were not replaced. Mice were randomly assigned to treatment groups as well as the order in which they received test agents. Experimenters were blinded to treatment groups whenever possible. However, because of differences in the solubility of several test agents, it was possible for the experimenter to differentiate vehicle from several test agents, even if it was not possible to distinguish the test agents from each other. No animals were eliminated from an experiment after the start of the experiment. Mice were used in this study as they express TRPM8 channels that contribute to nerve injury-induced cold hypersensitivity and the regulation of CBT. Mice also demonstrated peripheral neuropathy in response to the administration of chemotherapeutics with behavioural changes similar to those reported in humans.

### 2.11 | Animals

Male and female C57Bl6J (8–12 weeks of age) were obtained from Taconic (Germantown, USA) or Jackson (Bar Harbor) Laboratories. Mice were housed in the Association for Assessment and Accreditation of Laboratory Animal Care International (AAALAC) accredited University of Pittsburgh Animal Facility, a specific pathogen free facility, by sex (five per cage for males, four per cage for females), in high-efficiency particulate air (HEPA) filtered ‘shoe-box’ style containers with sterile wood chip bedding. Standard mouse chow and water were available ad libitum. Mice implanted with telemetry devices were housed individually. The facility is temperature and humidity controlled with lights on a 12:12 cycle (on at 7 AM). The temperature of the housing facility is maintained at 21°C and the humidity between 40% and 70%. Mice were acclimatised in the facility for 5–7 days prior to initiating any procedures. Behavioural experiments are performed within the animal facility and thermoregulation experiments are performed in the mouse home cages during the dark phase. Mice were humanely killed with CO_2_ asphyxiation followed by decapitation to ensure death, according to American Veterinary Medical Association approved methods. All procedures involving mice were approved by the University of Pittsburgh Institutional Animal Care and Use Committee (IACUC). Animal studies are reported in compliance with the ARRIVE guidelines (Percie du Sert et al., 2020) and with the recommendations made by the *British Journal of Pharmacology* (Lilley et al., 2020).

### 2.12 | Chemotherapy-induced peripheral neuropathy

Male and female C57 BL/6J mice from Taconic Labs were randomized by sex and cage to oxaliplatin or vehicle groups. Mice were weighed and injected intraperitoneally (i.p.) with either oxaliplatin (3 mg·kg^−1^), or vehicle (5% dextrose) once per day every day for five consecutive days, followed by five days off, and then by a second round of once per day for five more days. Therefore, mice received a total of 10 injections for a total of 30 mg·kg^−1^. The average weight of the females was 18 g while of the males was 20 g. Consequently, the females received an average of 540 and the males 600 μg in total. Behavioural testing was started after the second round of oxaliplatin/vehicle administration. The cold-plantar test was used to assess changes in cold sensitivity. A 3 ml syringe barrel filled with powdered dry ice was used as the stimulus. The dry ice is placed on the glass (borosilicate glass; thickness, 1/8 in. (3.17 mm); temperature, 75°F (24°C), measured with a calibrated IR temperature probe) beneath the hindpaw and the latency to withdraw was determined (Yilmaz et al., 2017). The baseline withdrawal latency was determined from the mean of three stimulus applications (at an inter-stimulus interval of ~5 min), while that at each time interval after the administration of a test agent was again the mean of three stimulus applications bracketing the time point in question (i.e., 5 min before, at the time point, and 5 min after). Mice in the two groups defined by oxaliplatin treatment were subsequently randomized to the order of treatment (with the TRPM8 antagonist VBJ103, or vehicle control). VBJ103 and vehicle solutions were administered subcutaneously (s.c.) at doses of 3, 10 and 30 mg·kg^−1^. Mice were tested every other day over 7 days so that each mouse received all four injections (vehicle and three doses of VBJ103). In a second cohort of male (n = 8) and female (n = 8) mice, all mice were treated with oxaliplatin as described above, and then randomized to the order of treatment with vehicle, VBJ103 (30 mg·kg^−1^) alone, the TRPA1 antagonist, HC-030031 (200 mg·kg^−1^) alone, or the combination of VBJ103 (30 mg·kg^−1^) and HC-030031 (200 mg·kg^−1^). A third cohort of male (n = 7) and female (n = 8) mice was also treated with oxaliplatin as described above. However, this cohort was subsequently randomized to the order of treatment with vehicle, VBJ103 (30 mg·kg^−1^) alone, the DAT inhibitor, GBR12909 (20 mg·kg^−1^) alone, or the combination of VBJ103 (30 mg·kg^−1^) and GRB12909 (20 mg·kg^−1^). All test agents in both follow-up cohorts were again administered in a volume of 5 ml·kg^−1^. Mice in both follow-up cohorts were again tested every other day over a period of 6 days. Finally, a fourth cohort of male (n = 4) and female (n = 4) mice was used to confirm that 200 mg·kg^−1^ HC-030031 was sufficient to block TRPA1. For this experiment, mice were randomized to the order of treatment with vehicle or HC-030031 (20 mg·kg^−1^). Thirty minutes after administration of vehicle or HC-030031, mice were injected with AITC (3 mg·kg^−1^ i.p.), and the number and duration of behaviours were quantified as described in Trevisan et al (Trevisan, Rossato, et al., 2013).

### 2.13 | Core body temperature (CBT) and activity measurements

Male and female C57Bl6J (Jackson Laboratories, Bar Harbor, USA, 8–12 weeks of age, 4 M:3F) were anaesthetised with isoflurane (2%–3% in 100% oxygen), and an HDX11 telemetry unit (Data Sciences) was inserted into the peritoneal cavity through a ventral midline incision. Animals were treated with buprenex (0.05 mg·kg^−1^, s.c.every 8 h for 72 h) and enrofloxacin (2 mg·kg^−1^, s.c.) and single-housed in a reverse light–dark cycle room (lights off 10 AM–10 PM). Mice were handled every day to acclimatise to injections, and experiments began 1 week later. All injections were performed between 1 and 2 PM. Testing was performed every other day with a single injection per day (5 ml·kg^−1^ body weight) of one of the following solutions: (1) VBJ103 (3, 10, 30, 100 or 300 mg·kg^−1^), (2) PBMC (30 mg·kg^−1^), (3) vehicle (5 ml·kg^−1^), (4) capsaicin (1 mg·kg^−1^) and (5) GBR12909 (20 mg·kg^−1^). All drugs and solutions were administered s.c. and i.p., but on different days, except capsaicin, GBR12909 and VBJ103 (100 and 300 mg·kg^−1^) which were only given s.c.. Body temperature was averaged into 5-min bins, whereas activity data were summed for the 5-min bin.

#### 2.13.1 | Intracerebroventricular (i.c.v.) administration

A second set of male C57Bl6J mice (Jackson Laboratories, n = 8) was implanted with telemetry as described above and a 26-gauge brain cannula (Plastics One) targeted at the lateral ventricle using coordinates relative to Bregma: 0.3 mm caudal, 1.0 mm lateral, 3.0 mm ventral. This second procedure was also performed under isoflurane anesthesia. The brain cannula was secured to the skull with two screws and dental cement. Animals were treated with buprenex (0.05 mg·kg^−1^, s.c. every 8 h for 48 h) and enrofloxacin (2 mg·kg^−1^, s.c.), singly housed and handled daily as described above. A single i.c.v. injection was performed every other day in a randomized order and included: (1) Galanin-like peptide (3 nmol/1 μL), (2) VBJ103 (306 nmol/1 μL) and (3) VBJ103 vehicle (1 μL). Galanin-like peptide produced a decrease in body temperature in five of eight mice. Therefore, body temperature data in response to VBJ103 and vehicle are presented in these five mice. These studies were carried out with a single dose of VBJ103 (306 nmol/1 μL or ~4 mg·kg^−1^), which was the highest dose achievable due to constraints of the injection volume and the solubility of VBJ103.

### 2.14 | Determination of brain levels in vivo

VBJ103 levels in the brain were determined by PsychoGenics Inc (Paramus, USA). VBJ103 and vehicle solutions were administered subcutaneously at a dose of 100 mg·kg^−1^. Male and female Sprague–Dawley rats (200–250 g) supplied by Envigo were used in this study and housed three per cage (26 cm × 47.6 cm × 20.3 cm) upon arrival. Rats with jugular vein catheters (JVC) were housed two per cage with a divider to prevent damage to catheters. Cages contained BedO’-Cob^®^ bedding with Nylabones^®^ for enrichment. Rats were acclimatised for 2 to 7 days prior to commencing studies. All rats were examined, handled and weighed prior to initiation of the study to assure adequate health and suitability. During the course of the study, 12/12 light/dark cycles were maintained with lights on at 6 AM. The room temperature was maintained between 20°C and 23°C with a relative humidity maintained around 50%. Chow (Lab diet 5001, LabDiet, St. Louis, MO, USA) and water were provided ad libitum throughout the acclimatisation period and during the treatment and collection period. Rats were randomly assigned to the experimental groups.

UPLC-MS/MS analysis was performed on a ODS 3 Inertsil C18 column 2.1 × 50 mm, 2 μm (GL Sciences 5020–84652), using a gradient of 40:60 solvent A (H_2_O + 0.5% formic acid):solvent B (ACN + 1.0% formic acid) → 0:100 for 2 min at a flow rate of 0.5 ml·min^−1^. The following parameters were used: injection volume 5 μl, column temperature 40°C, pre-column temperature 40°C and sample temperature 4°C. Eluted peaks were monitored with a Waters Xevo TQ-XS coupled to an Acquity i-Class Plus UPLC setup (range: 334.19 Da to 155.04 and 198.08 Da). R_T_ = 1.15 min. The range of quantitation was 0.25 to 10,000 ng·ml^−1^ for brain. The software Masslynx V4.2 SCN1012 was used for instrument control and data acquisition.

#### 2.14.1 | Sample collection

Brain collection was performed via live decapitation. Whole brains were collected at 1 h following administration and upon completion of serial collections (24 h). Resultant brains were rinsed in saline and frozen immediately on a cold plate (−77°C) before placement into a pre-weighed specimen container. Brain weights were then determined prior to placement of the samples on dry ice followed by relocation to a −80°C freezer pending bioanalysis.

#### 2.14.2 | Sample preparation

Pre-weighed whole brain tissue was homogenized (Omni Prep multichannel tissue homogenizer) using a 1:1 MilliQ water (containing 0.2% acetic acid)/acetonitrile mixture which was added to the brain using a 4:1 solution to brain ratio. Subsequently, the homogenate was centrifuged for 10 min at 4°C at a speed of 2688 *g*. Three 25-μl aliquots out of the top layer of the centrifuged homogenate were crashed into three QuanRecovery wells, each containing 150 μl of acetonitrile/internal standard. The crash was then centrifuged for 20 min at 2688 *g* while maintaining a temperature of 4°C. Mixing of the resultant supernatant with water (3:2) provided samples for triplicate analysis. Brain standards, quality controls (QCs) and blanks were prepared using a male Sprague–Dawley brain acquired from BioIVT using the same protocol outlined above. All standards, QCs, samples and blanks were then loaded onto the UPLC-MS/MS for analysis.

#### 2.14.3 | Determination of brain concentration

The concentration of VBJ103/mass was based on brain weights. The volume of the homogenate was based on a 4:1 dilution of the brains. The volumes of homogenate was calculated based on brain weights in Table S13.

### 2.15 | Statistical analysis

The data and statistical analysis comply with the recommendations of the *British Journal of Pharmacology* on experimental design and analysis in pharmacology (Curtis et al., 2022). Group sizes were determined by power analysis based on variance established from previous studies, with an *F*-test (ANOVA with either main effects and interactions, or repeated measures within-between interactions) and a moderate effect size, a power of 0.8, and alpha of 0.05. This *P* value was maintained for all analyses. For all experiments, all data were included. Raw and transformed data were analysed with two- and three-way analyses of variance (ANOVAs) with SigmaPlot (V14, Systat Software Inc) and/or SPSS (V28.0.1.1 (14), IBM SPSS Statistics). Data were first assessed for normality (Shapiro–Wilk) and equal variance (Brown–Forsythe). Post-hoc tests (Holm-Sidak unless otherwise indicated) were run only if F achieved P<0.05 for interactions and there was no significant variance inhomogeneity.

### 2.16 | Nomenclature of targets and ligands

Key protein targets and ligands in this article are hyperlinked to corresponding entries in the IUPHAR/BPS Guide to PHARMACOLOGY http://www.guidetopharmacology.org and are permanently archived in the Concise Guide to PHARMACOLOGY 2023/23 (Alexander, Christopoulos et al., 2023; Alexander, Fabbro et al., 2023; Alexander, Mathie et al., 2023).

## 3 | RESULTS

### 3.1 | In vitro selectivity and safety profiling of the TRPM8 antagonist VBJ103

**FIGURE 1:**
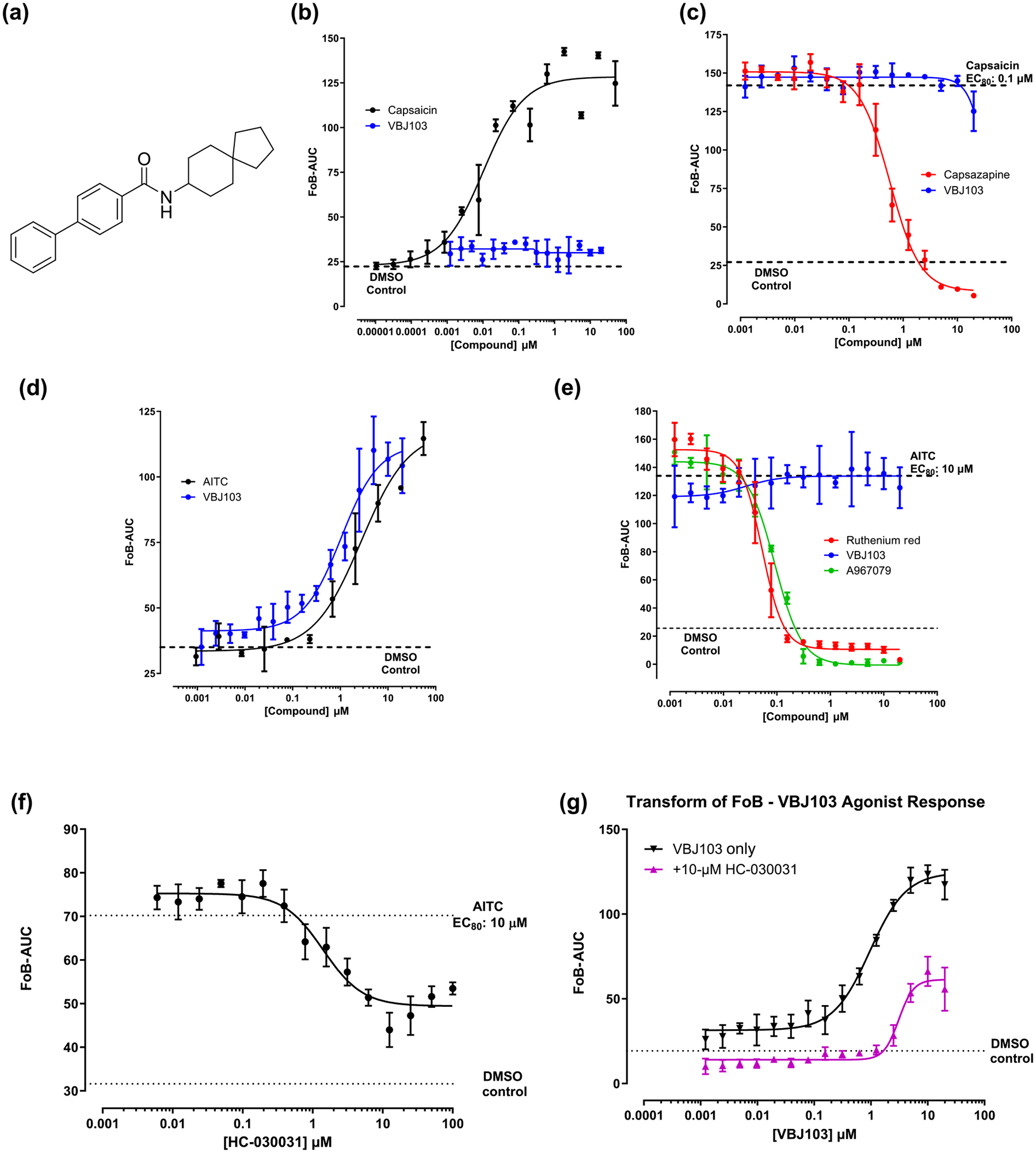
Selectivity profiling of VBJ103 at human TRPV1 and TRPA1 channels, determined in Flexstation Ca^2+^ flux assays in HEK-293 transfected cells. (a) Chemical structure of VBJ103 (N-(spiro[4.5]decan-8-yl)-[1,1′-biphenyl]-4-carboxamide). (b, c) agonist and antagonistactivity of VBJ103 at TRPV1. Concentration–response analysis of capsaicin activation and casazepine inhibition are shown. Capsaicin TRPV1 EC_50_: 10.3 ± 7.5 nM; capsazepine TRPV1 IC_50_: 0.566 ± 0.086 μM. (d, e) agonist and antagonist activity of VBJ103 at TRPA1. Concentration–response analysis of AITC activation and A-967079 and ruthenium red inhibition are shown. AITC TRPA1 EC_50_: 2.75 ± 1.89 μM; A-967079 TRPA1 IC_50_: 91 ± 11 nM; ruthenium red TRPA1 IC_50_: 53 ± 7 nM. (f) Concentration-dependent inhibition by the TRPA1 antagonist HC-030031 in TRPA1 cells activated by AITC. HC-030031 TRPA1 IC_50_: 2.00 ± 0.834 μM. (g) Concentration–response of HC-030031 inhibition of VBJ103-induced Ca^2+^ flux. VBJ103 TRPA1 EC_50_: 0.955 ± 0.210 μM; VBJ103 TRPA1 EC_50_ in the presence of HC-030031: 3.09 ± 0.592 μM. AUC, area under curve; FoB, fold over baseline.

We confirmed the structure of the spiro[4.5]decan-8-yl analogue VBJ103 (Figure 1a) using X-ray crystallography (Figure S1 and Tables S1–S7). We previously demonstrated ~10-fold selectivity for the heterologously expressed human TRPM8 versus TRPA1 and TRPV1 (Journigan et al., 2020). We extend these initial observations to assess agonist and antagonist activity at both subtypes across a wider concentration range (Figure 1). The EC_50_ of the TRPV1 agonist, capsaicin, was 10.3 ± 7.5 nM. The IC_50_ of the TRPV1 antagonist capsazepine was 0.566 ± 0.086 μM against an EC_80_ concentration (0.1 μM) of capsaicin (Figure 1b,c). In contrast, VBJ103 alone exhibited no apparent agonist activity at TRPV1. Nor was there a detectable inhibition of the Ca^2+^ signal evoked with an EC_80_ concentration (0.1 μM) of capsaicin (Figure 1b,c). The EC_50_ of the TRPA1 agonist AITC was 2.75 ± 1.89 μM. The IC_50_’s of the selective TRPA1 antagonist A-967079 (Chen et al., 2011; Ye et al., 2021) and the non-specific antagonist ruthenium red against an EC_80_ concentration (10 μM) of AITC were 91 ± 11 nM and 53 ± 7 nM, respectively (Figure 1e). The IC_50_ of HC-030031 (Eid et al., 2008; McNamara et al., 2007) against an EC_80_ concentration (10 μM) of AITC was 2.00 ± 0.83 μM (Figure 1f). HC-030031 achieved a maximum inhibition of 54%. VBJ103 exhibited agonist activity at TRPA1, with an EC_50_ of 1.04 ± 0.48 μM (Figure 1d). A saturating concentration of HC-030031 (10 μM) had a modest influence on the potency of VBJ103 (3.2-fold shift in EC_50_, from 0.96 ± 0.21 to 3.09 ± 0.59 μM) and significantly reduced the maximum efficacy to 51%, respectively, consistent with noncompetitive antagonism (Figure 1g). However, VBJ103 exhibited no detectable antagonist activity at TRPA1 against an EC_80_ concentration (10 μM) of AITC (Figure 1e).

**FIGURE 2:**
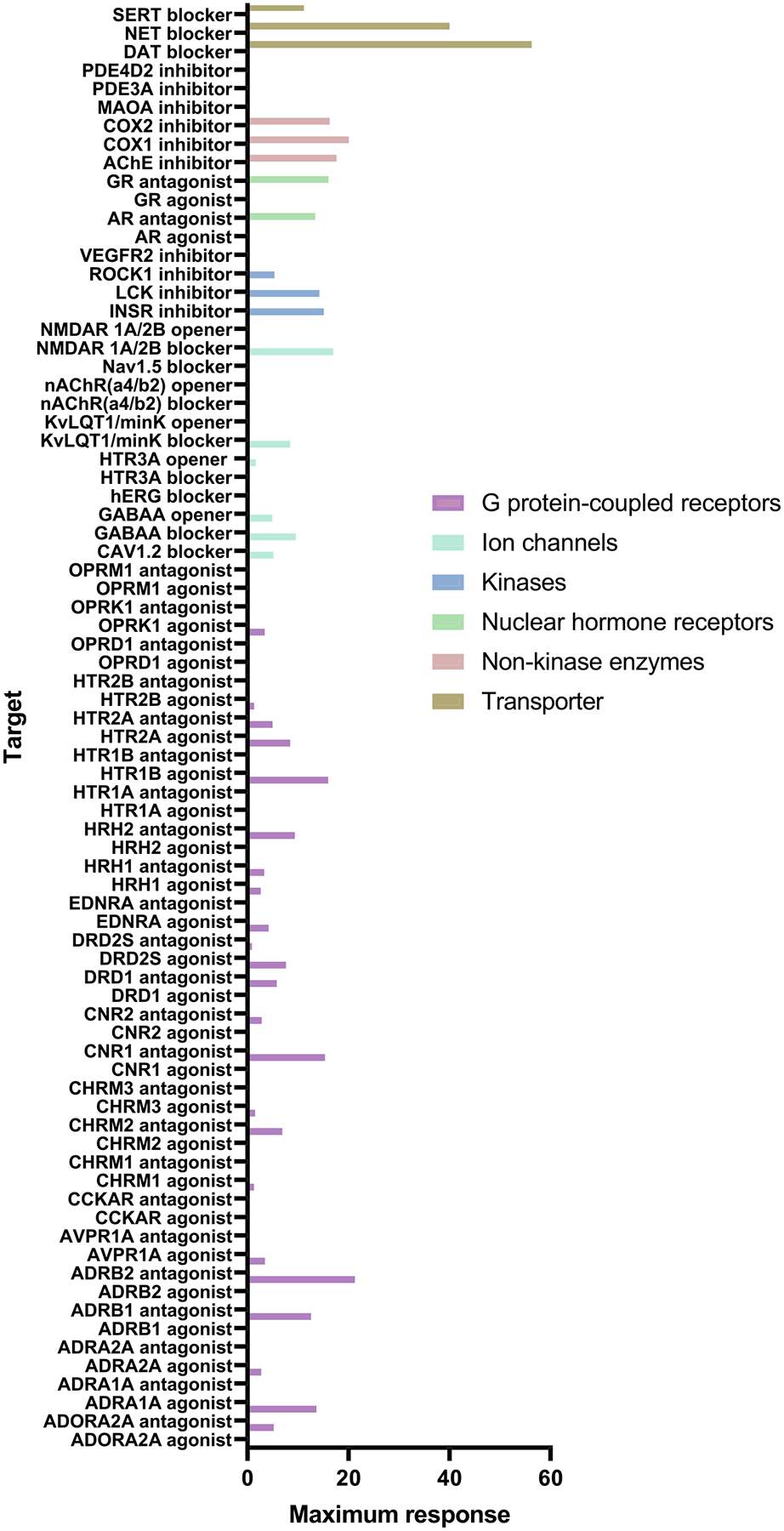
In vitro safety profiling of VBJ103 (100 μM) across 78 recombinant human targets. Data represent a single experiment.

Screening across a broad panel of targets (Bowes et al., 2012) was performed at 10 concentrations of VBJ103, from 0.005 to 100 μM. Inhibition of the dopamine transporter DAT was the only dose-dependent effect detected for VBJ103, with an IC_50_ of 125 nM and a maximal inhibition of ~60% (Figure S3). VBJ103 had no activity at the μ, δ and κ opioid receptors (OPRM1, OPRD1 and OPRK1) (Figures 2 and S2 and Table S9). VBJ103 showed no activation of opioid receptors when tested in positive allosteric modulator mode (in the presence of an EC_20_ of the opioid receptor agonist) at 10 concentrations in duplicate per target, from 0.001 to 100 μM (Figure S5 and Table S10). Together, our data demonstrate that VBJ103 is highly selective for TRPM8 with few potential off-target effects.

### 3.2 | Antinociceptive effects against noxious cold in oxaliplatin-treated mice with VBJ103 administration

**FIGURE 3:**
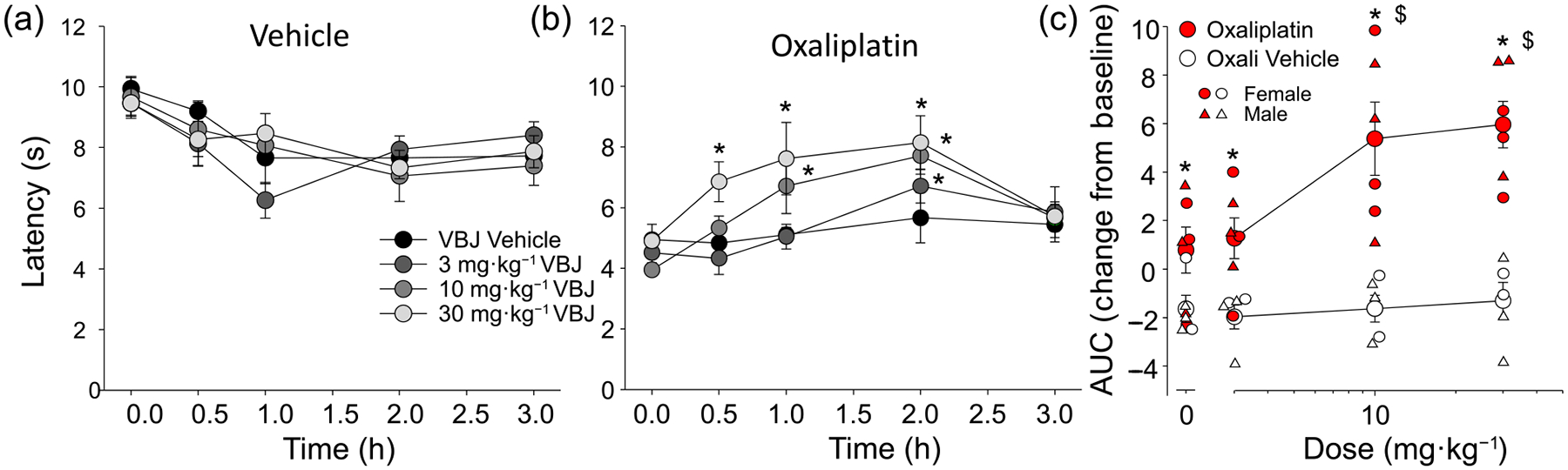
Inhibition of oxaliplatin-induced cold hyperalgesia by the TRPM8 antagonist VBJ103 (VBJ). (a) Changes in cold sensitivity as assessed with the cold-plantar test in mice receiving oxaliplatin vehicle (Vehicle, 5% dextrose, n = 5), following subcutaneous injection of VBJ103 vehicle (VBJ Vehicle), or one of three doses of VBJ103. (b) Mice randomized to the Oxaliplatin group (n = 7) were tested in parallel with the mice in (a), following two rounds of oxaliplatin administration. Cold hypersensitivity is evident with the shorter withdrawal latency in this group at baseline. Each mouse in (a) and (b) received all four injections in a random order in testing over 6 days. (c) Data analysed as an area under or over the curve (AUC) defined by the change in withdrawal latency from baseline over time. Data were analysed by three-way ANOVA (a + b) or two-way ANOVA (c), followed by Holm–Sidak test. In (b), * is *P* < 0.05 relative to vehicle. In (c), * are significant (*P* < 0.05) differences between treatment groups, while $ are within group comparisons (*P* < 0.05) relative to baseline. AUC, arera under curve.

We previously demonstrated that intraplantar administration of VBJ103 dose-dependently suppresses oxaliplatin-induced hypersensitivity to non-noxious cooling (acetone drop) (Journigan et al., 2020). However, systemic administration would be more appropriate for more widespread cold hypersensitivity. The partition coefficient (logD) of VBJ103 was 3.21 (Table S11), which is sufficiently lipophilic (Di & Kerns, 2016) to support further studies in mice receiving a systemic dose administered s.c. (between the shoulder blades). VBJ103 has a T_1/2_ in mouse liver microsomes of 30 min (Journigan et al., 2020, and Table S11), suggesting we should be able to detect an antinociceptive effect at this time-point. Consequently, the withdrawal latency to noxious cold stimulation of the glabrous skin of the hindpaw was measured before and after (30, 60, 120 and 180 min) drug administration (Figure 3). To rule out a test day-dependent change in baseline withdrawal latency, baseline data were analysed with a two-way ANOVA, which revealed a main effect of group (oxaliplatin vs. vehicle treated animals), but no significant influence of test day (not shown). Raw data were analysed with a three-way RM ANOVA that included group (2) × drug (4) × time (5). There were significant interactions between all three factors (*P* < 0.05). We have highlighted the within oxaliplatin group effects: significant reversals in hypersensitivity observed with the 30 mg·kg^−1^ dose (at 0.5, 1 and 2 h), the 10 mg·kg^−1^ dose (at 1 and 2 h) and the 3 mg·kg^−1^ dose (at 2 h). While both male and female mice were used in the cohort used for this dose–response study, the study was not powered to detect a sex difference. That said, there was no evidence of even a trend in either the magnitude of the oxaliplatin-induced hypersensitivity or the influence of VBJ103 (Figure 3c). While not highlighted with stars, there was also a significant time effect in the non-oxaliplatin treated animals, such that all animals exhibited a significant decrease in latency over time relative to baseline. This effect was not dose-dependent however. To clearly illustrate the interaction between drug and time, data were analysed as an area under the curve (AUC, Figure 3c). Data transformed in this manner were analysed with a two-way ANOVA which also resulted in a significant interaction between group and drug treatment (*P* < 0.05). Post hoc analysis confirmed significant differences between treatment groups at baseline and at all drug doses. More importantly, in the oxaliplatin (but not non-oxaliplatin group), the increase in latency with the 10 and 30 mg·kg^−1^ groups was significant compared to baseline (*P* < 0.05). These results confirm that systemic administration of VBJ103 significantly attenuates chemotherapy-induced peripheral neuropathy (CIPN)-induced cold hypersensitivity.

**FIGURE 4:**
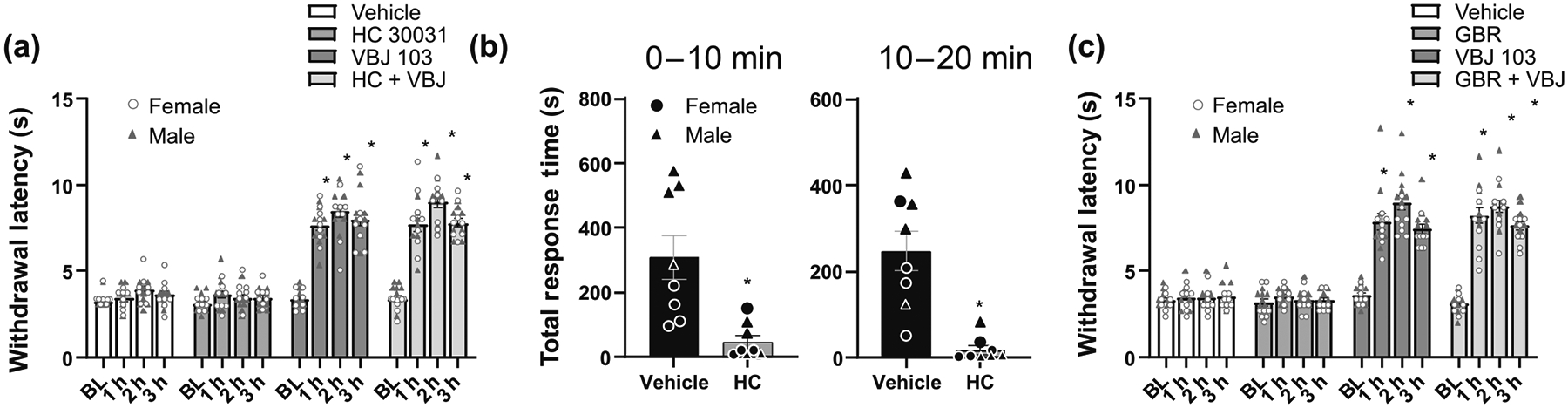
The antinociceptive effects of VBJ103 (VBJ) against oxaliplatin-induced cold hypersensitivity are independent of TRPA1 and dopamine transporter (DAT) inhibition. (a) Changes in cold sensitivity as assessed with the cold-plantar test in mice receiving vehicle (0.5% Methocel), the TRPA1 antagonist HC-030031 (HC) (200 mg·kg^−1^), VBJ103 (30 mg·kg^−1^) or a combination of HC-030031 and VBJ103. Baseline thresholds were measured before injection in each of the four groups. n = 16, 8M:8F. (b) Total response time of AITC-treated mice, quantified over 20 min after subcutaneous injection of vehicle (0.5% Methocel) or the TRPA1 antagonist HC-030031. n = 8, 4M:4F. (c) Changes in cold sensitivity as assessed with the cold-plantar test in mice receiving vehicle (1:1 DMSO:water), the DAT inhibitor GBR12909 (20 mg·kg^−1^), VBJ103 (30 mg·kg^−1^) or a combination of VBJ103 and GBR12909. Baseline thresholds were measured before injection in each of the four groups. n = 15, 7M:8F. Data were analysed by three-way ANOVA (a + c) or two-way ANOVA (b), followed by Holm–Sidak test. * is *P* < 0.05 relative to vehicle.

We next confirmed the antinociceptive effects of VBJ103 are not due to TRPA1 antagonist activity not captured in our in vitro assay. In a second cohort of male (n = 8) and female (n = 8) mice treated with oxaliplatin, mice were randomized to the order of treatment with vehicle, VBJ103 (30 mg·kg^−1^) alone, the TRPA1 antagonist HC-030031 (Eid et al., 2008) (200 mg·kg^−1^) alone, or the combination of VBJ103 (30 mg·kg^−1^) and HC-030031 (200 mg·kg^−1^). Interestingly, we failed to detect an influence of HC-030031 on oxaliplatin-induced cold hypersensitivity (Figure 4a). However, consistent with our initial dose–response study, VBJ103 alone significantly attenuated oxaliplatin-induced cold hypersensitivity at all time points tested (*P* < 0.05 vs. baseline as well as vehicle). Furthermore, the influence of the combination of VBJ103 and HC-030031 on the withdrawal latency to noxious cold stimuli was not detectably different from the influence of VBJ103 alone. Of note, this second cohort of animals was powered to detect the presence of a sex difference with at least a moderate effect size. But as with the first cohort, there was no evidence of a trend for a sex difference in either hypersensitivity associated with oxaliplatin, or the antinociceptive effects of VBJ103. To confirm that the dose of HC-030031 used in the antinociception study was sufficient to block TRPA1, we assessed the impact of HC-030031 (200 mg·kg^−1^) on the nociceptive behaviours evoked with an i.p. injection of AITC (3 mg·kg^−1^). Consistent with previous results (Trevisan, Rossato, et al., 2013), AITC-induced nociceptive behaviours were significantly attenuated by HC-030031 (Figure 4b, *P* < 0.05). Despite the small group size, a significant (*P* < 0.05) sex difference was detected both in the duration of pain behaviours evoked with AITC and in the magnitude of the HC-030031 mediated inhibition of the behaviours. These results suggest that TRPA1 is not responsible for the antinociceptive effect of VBJ103.

Given evidence of VBJ103 activity at the dopamine transporter, combined with evidence that dopamine may contribute to the inhibition of cold hypersensitivity (Cobacho et al., 2014; Lancon et al., 2021), we next assessed the impact of DAT inhibition on oxaliplatin-induced cold hypersensitivity. A third cohort of male (n = 7) and female (n = 8) mice all treated with oxaliplatin was again randomized to the order of treatment with vehicle, VBJ103 30 mg·kg^−1^ alone, the DAT inhibitor GBR12909 (20 mg·kg^−1^; Novick et al., 2015; Pozzi et al., 1994), alone or the combination of VBJ103 and GBR12909. Consistent with previous results suggesting that 20 mg·kg^−1^ GBR12909 is sufficient to increase dopamine in the brain, we were able to detect a small but significant increase in activity in mice that received this dose of the drug (see below). However, there was no detectable influence of GBR12909 alone on the withdrawal latency to noxious cold stimuli. In contrast, and consistent with the two previous cohorts of mice, VBJ103 alone significantly reversed the oxaliplatin-induced cold hypersensitivity (*P* < 0.05). Furthermore, there was no detectable influence of GBR12909 on the withdrawal latency when administered in combination with VBJ103 (Figure 4c). As with the previous cohorts, there was no evidence of a sex difference in either the magnitude of the oxaliplatin-induced hypersensitivity, or the antinociceptive influence of VBJ103. Together, these results suggest that DAT inhibition does not contribute to VBJ103-mediated antinociception in oxaliplatin treated mice.

### 3.3 | Effects of VBJ103 on core body temperature (CBT) and activity

**FIGURE 5:**
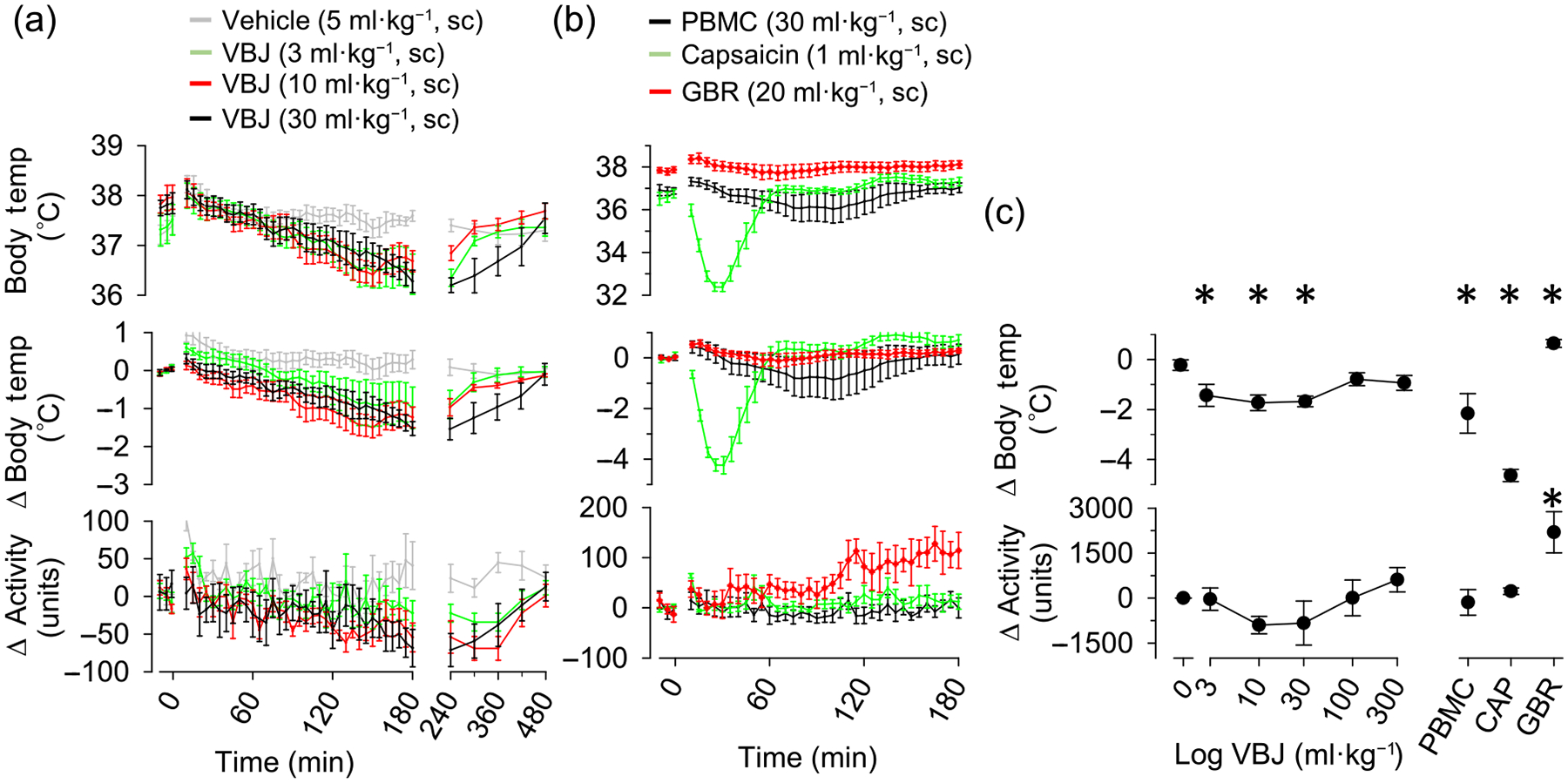
Effect of subcutaneous VBJ103 on core body temperature. Mean ± SEM of core body temperature, change in core body temperature, or home cage activity of mice (n = 7, 4M:3F) injected with (a) vehicle (5 ml·kg^−1^, s.c.) or VBJ103 (3, 10 and 30 mg·kg^−1^, s.c.) and (b) the TRPM8 antagonist PBMC, capsaicin and the DAT inhibitor GBR 12909. (c) VBJ103 dose–response as a function of maximum drop in core body temperature. Data were analysed by a mixed ANOVA with independent *t*-tests with layered Bonferroni correction (*P < 0.05 versus vehicle).

To test whether VBJ103 affects CBT, a separate group of mice was implanted with telemetry units to measure CBT after s.c. and i.p. administration of VBJ103. Since there were no differences between male and female mice with respect to baseline or test-agents effects on CBT, the results were combined. VBJ administered subcutaneously (3, 10 and 30 mg·kg^−1^) produced a progressive fall in CBT (1–2°C) over 180 min without any significant change in home cage activity (Figure 5a). The peak drop in CBT was significant (*P* < 0.05), compared to vehicle treated animals. The drop in CBT resolved for all doses of VBJ103 (3, 10 and 30 mg·kg^−1^), returning to baseline values after 223 ± 25 min, 262 ± 48 min and 380 ± 59 min, respectively. Higher doses of VBJ103 (100 and 300 mg·kg^−1^, s.c.) did not alter CBT or home cage activity compared to vehicle treated animals (*P* > 0.05). The TRPM8 antagonist PBMC (30 mg·kg^−1^) administered s.c. decreased CBT (1–2°C) with a peak between 60 and 120 min without changes in home cage activity. This change in CBT was significantly greater than that associated with vehicle (*P* < 0.05). As a positive control, the TRPV1 agonist capsaicin produced a significantly larger drop in CBT with a peak within 60 min than either s.c. VBJ103 or PBMC. Finally, to rule out the possibility that an increase in activity could mask a drug-induced drop in CBT, we assessed the impact of GBR12909 on activity and CBT. Consistent with this possibility, GBR12909 was associated with an increased body temperature (*P* < 0.05) and home cage activity (*P* < 0.05) relative to vehicle treated animals.

**FIGURE 6:**
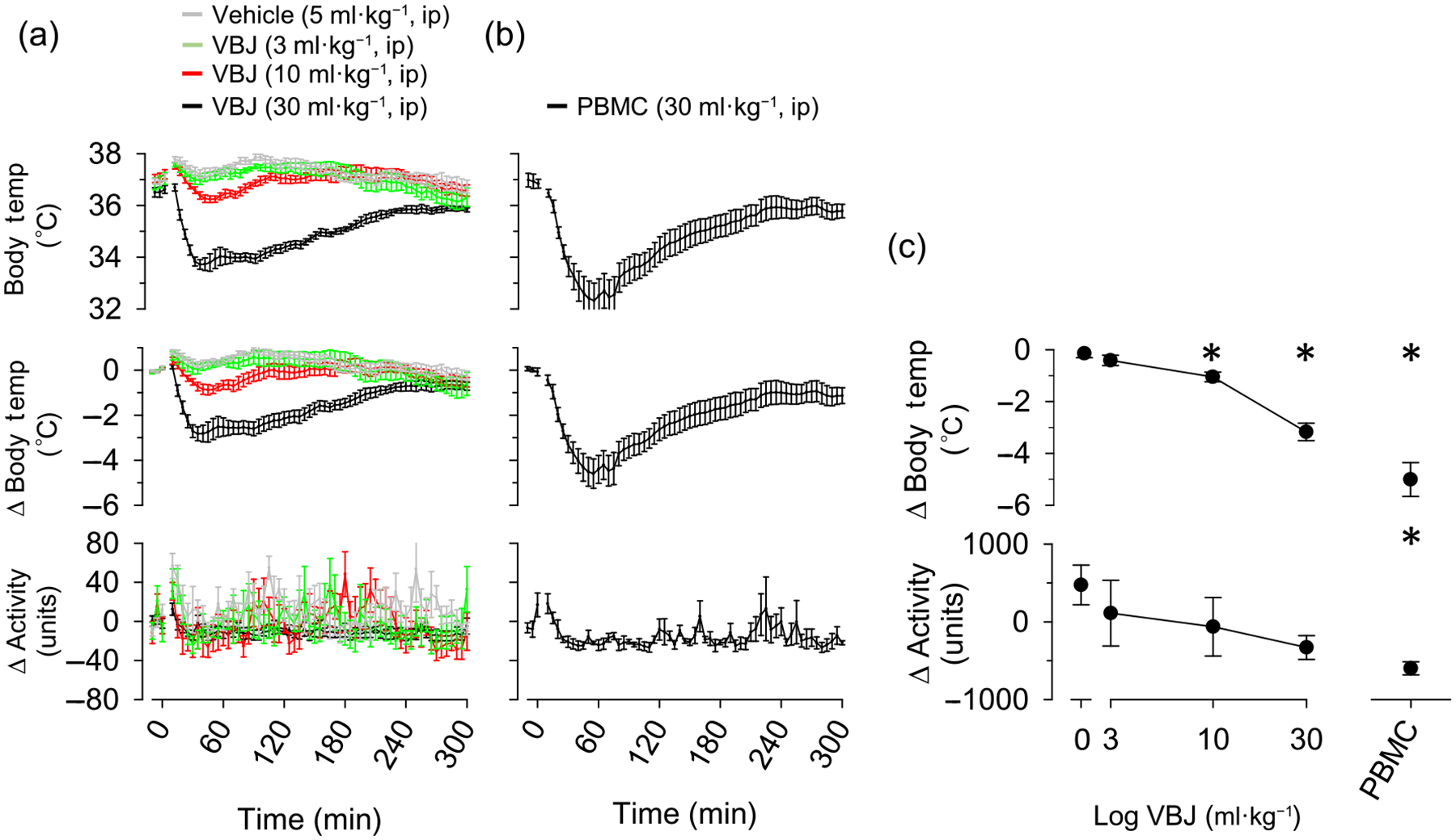
Effect of intraperitoneal VBJ103 (VBJ) on core body temperature. Mean ± SEM of core body temperature, change in core body temperature or home cage activity of mice (n = 7; 4M:3F) injected with (a) vehicle (5 ml·kg^−1^, i.p.), VBJ103 (3, 10 or 30 mg·kg^−1^) or (b) the TRPM8 antagonist PBMC. (c) VBJ103 dose–response as a function of maximum drop in core body temperature. Data were analysed by a mixed ANOVA with independent *t*-tests with layered Bonferroni correction. *P < 0.05 versus vehicle.

Since PBMC administered i.p. was previously reported to produce much larger drops in CBT (~5°C) (Knowlton et al., 2011), we sought to determine whether route of administration might influence the impact of TRPM8 antagonists on CBT. VBJ103 administered i.p. produced a dose-dependent fall in CBT that peaked within 60 min but with no detectable change in home cage activity (Figure 6a). The drop in CBT in response to 10 and 30 mg·kg^−1^ VBJ103 was significantly greater than to 3 mg·kg^−1^ VBJ103 and vehicle. The drop in CBT was not different between vehicle and 3 mg·kg^−1^ VBJ103. No further drop in CBT was observed up to 6 h with 30 mg·kg^−1^ VBJ103 (data not shown). Consistent with these previous results, i.p. administration of PBMC also produced a ~5°C fall in CBT that peaked within 60 min and caused parallel decrease in home cage activity (Figure 6b).

### 3.4 | Intracerebroventricluar (i.c.v) administration of VBJ103 does not impact CBT

**FIGURE 7:**
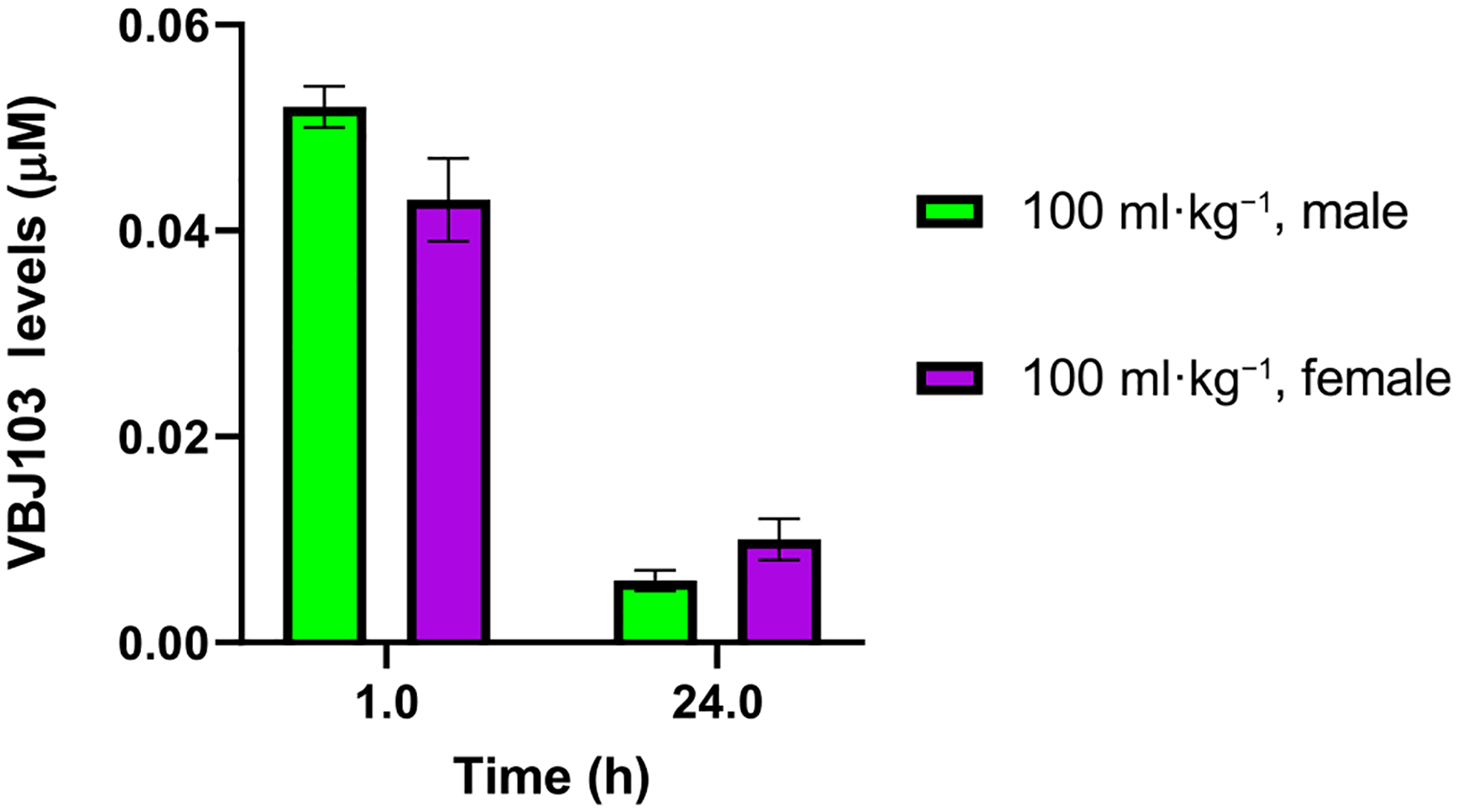
Brain levels of VBJ103 in male and female Sprague–Dawley rats following s.c. injection (100 mg·kg^−1^), at 1 and 24 h post injection as measured by UPLC-MS/MS analysis. Data points are mean values ± SEM (n = 3 for males and females at 1 h, and n = 4 rats for males and females at the 24-h timepoint). As n=3 or n=4 for these experiments, statistical analysis was not carried out, and results should be regarded as preliminary.

**FIGURE 8:**
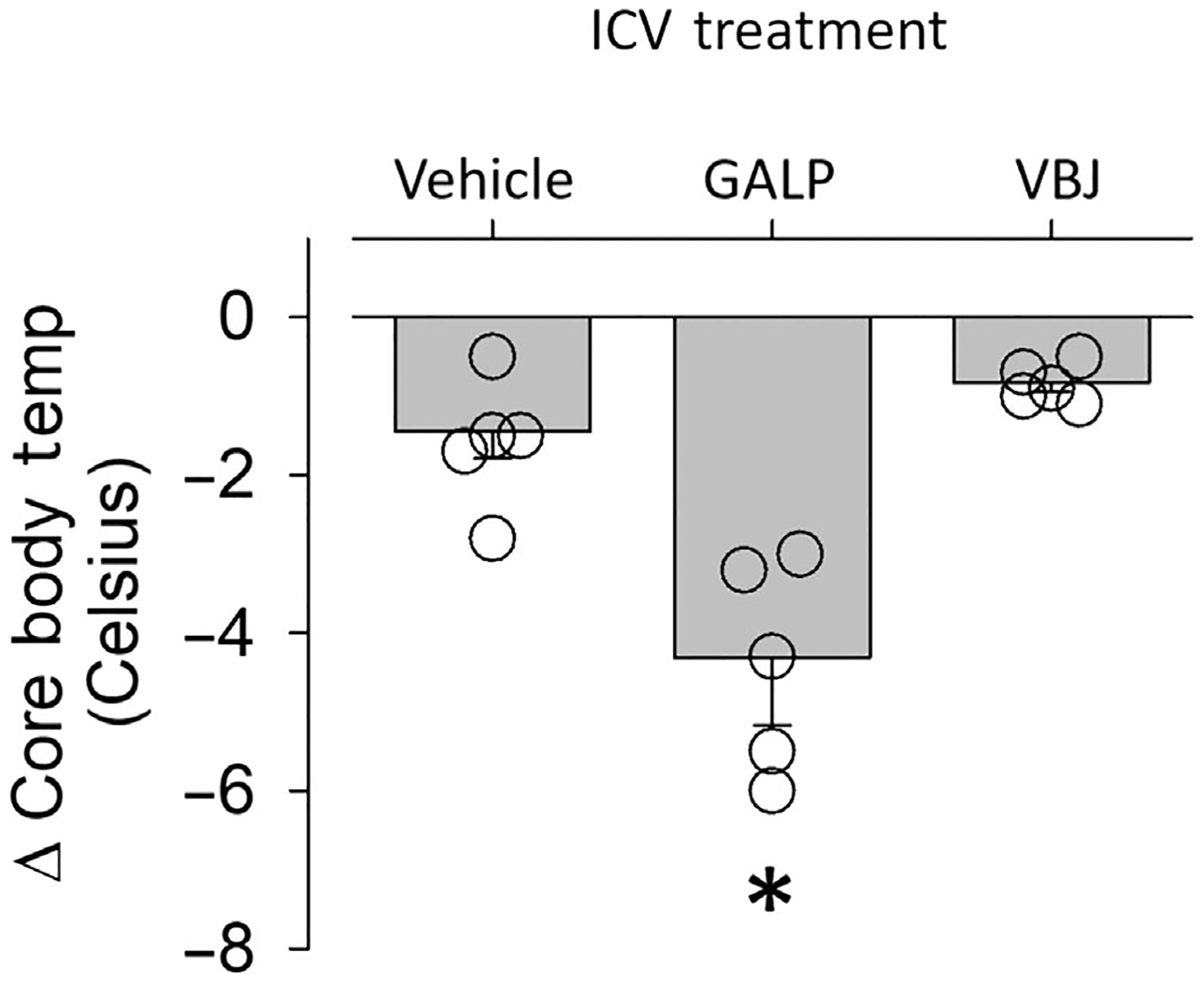
Intracerebroventricular (i.c.v.) administration of VBJ103 (VBJ) does not affect core body temperature. Mean ± SEM and individual data points of the peak decrease in core body temperature after i.c.v. injection (1 μl) of DMSO (vehicle), Galanin-like peptide (GALP, 3 nmol) and VBJ103 (306 nmol, i.c.v). *P < 0.05 GALP versus DMSO and VBJ103, analysed by repeated measures (RM) one-way ANOVA with Tukey post hoc test. Baseline body temperature did not differ between treatment groups (vehicle: 36.7 ± 0.2 vs. GALP: 37.1 ± 0.2°C).

Given evidence of a role for centrally expressed TRPM8 in the regulation of CBT (Tsuneoka et al., 2023), we tested whether VBJ103 gets into the CNS with s.c. administration. These studies were carried out in rats as part of the NINDS Preclinical Screening Platform for Pain (PSPP) program, prior to initiating the other in vivo studies. Mean brain concentrations of VBJ103 (100 mg·kg^−1^, s.c.) in male and female rats were 52 ± 2 nM and 43 ± 4 nM, respectively (Figure 7). These concentrations should be high enough to block TRPM8 receptors in the CNS based on our previously reported IC_50_ of VBJ103 (64 ± 2 nM) against menthol-stimulated currents (Journigan et al., 2020). Since potential species differences may influence drug pharmacokinetics, we directly tested whether centrally administered VBJ103 decreased CBT in mice. A second set of mice was instrumented with telemetry devices and a lateral ventricle brain cannula. Intracerebroventricular infusion of galanin-like peptide (3 nmol in 1 μL) decreased CBT (>3°C) within 60 min in five of eight mice tested (Figure 8). Intracerebroventricular injection of VBJ103 (306 nmol per 1 μL) did not significantly (*P* > 0.05) alter CBT compared to mice injected with vehicle (Figure 8).

## 4 | DISCUSSION

We hypothesized that residues on TRPM8 associated with the regulation of environmental cold and CBT may be spatially distinct, suggesting it is possible to block environmental cold-activation of TRPM8, without influencing CBT (i.e., thermoregulation). To test this hypothesis, we assessed the impact of systemic and i.c.v. administration of VBJ103 on oxaliplatin-induced cold hypersensitivity and CBT. We first confirmed the selectivity of VBJ103, as well as its safety profile. VBJ103 exhibited high selectivity over TRPV1 and TRPA1. It did demonstrate low potency (EC_50_ > 1 μM) as an agonist of TRPA1. Safety profiling did not reveal any liabilities, except for partial inhibition at the dopamine transporter DAT at relatively low concentrations (IC_50_ ~ 125 nM). We next demonstrated that VBJ103 can dose-dependently (3 to 30 mg·kg^−1^) suppress oxaliplatin-induced hypersensitivity to cold stimuli for up to 3 h post administration; however, baseline responses to noxious cold were not altered with VBJ103 treatment compared to vehicle. This antinociceptive effect was not dependent on TRPA1 or DAT. VBJ103 delivered s.c. decreased CBT by 2°C over the same time course at which antinociception was observed. Temperature changes gradually returned to baseline values. In contrast, i.p. administration of VBJ103 dose-dependently decreased CBT. No effect on CBT was observed with i.c.v. administration of VBJ103.

The selectivity and safety profile of VBJ103 continue to look promising. We detected no measurable inhibitory effects of VBJ103 at TRPV1 or TRPA1 in transfected HEK-293 cells across a large concentration range. However, we did detect two off-target effects: agonist activity at TRPA1 and antagonist activity at DAT, although with relatively low potency and efficacy at either. We were unable to detect evidence of VBJ103 activity at either TRPA1 or DAT in vivo however. With respect to DAT, VBJ103 was associated with no detectable increase in mouse activity, a hallmark of a DAT inhibition-induced increase in dopamine levels, even at very high systemic concentrations of VBJ103. Furthermore, given that an increase in dopamine is antinociceptive, the antinociceptive effects of the combination of VBJ103 and GBR12909 should have been greater than those associated with VBJ103 alone; this was not detected either. Nevertheless, any activity at DAT is a potential concern if VBJ103 was used in combination with medications designed to manipulate dopamine signalling.

The observation that VBJ103 activates TRPA1 in vitro raises the possibility that the antinociceptive effects of VBJ103 were due to the activation and subsequent desensitization of TRPA1, rather than a block of TRPM8. Consistent with this possibility, there is evidence that TRPA1 agonists such as cinnamaldehyde inhibit cold responses in humans (Namer et al., 2005; Weyer-Menkhoff & Lotsch, 2019). Arguing against this possibility, however, are the following observations. First, while not systematically studied, we detected no evidence of even transient activation of TRPA1 with VBJ103, whether administered s.c. or i.p., which should have been associated with overt nociceptive behaviours such as vocalization, licking, or biting the injection site following s.c. administration, or stretching behaviours such as those evoked with AITC when administered i.p.; however, no such behaviours were observed. Second, and consistent with this suggestion, there was no evidence of even a transient change in activity, increase or decrease, that differed from vehicle treated animals, in the telemetry studies. Third, while HC-030031 was only able to antagonize ~50% of the AITC evoked response in vitro, the antagonism of AITC evoked nociceptive behaviour was still readily detectable. And fourth, HC-030031 failed to influence the antinociceptive effects of VBJ103. That is, if activation, and subsequent desensitization of TRPA1 contributed to the antinociceptive effects of VBJ103, VBJ103-induced antinociception should have been attenuated by prior treatment with HC-030031. Thus, we suggest the more likely interpretation of our data is that the antinociceptive effects of VBJ103 are solely due to the inhibition of TRPM8. Consistent with this possibility, we previously demonstrated that VBJ103 (30 mg·kg^−1^, s.c.) suppresses icilin-induced wet dog shaking (WDS) behaviour (Journigan et al., 2020), a behaviour dependent on the presence of TRPM8 as indicated by the loss of wet dog shaking in TRPM8 knock-out mice (Colburn et al., 2007; Dhaka et al., 2007). Nevertheless, given that HC-030031 only antagonized ~50% of the VBJ103-induced activation of TRPA1 in vitro, this conclusion is made with caution.

Our behavioural results are partially consistent with our initial hypothesis, with respect to the ability to block environmental cold-activation of TRPM8 in vivo. The antinociceptive effects of VBJ103 are in line with prior studies demonstrating antinociceptive effects of TRPM8-selective antagonists in this model (Bertamino et al., 2018, 2020; Bonache et al., 2020; Chaudhari et al., 2013; Iraci et al., 2022; Martin-Escura et al., 2023, 2022; Mizoguchi et al., 2016). There was no apparent sex difference in efficacy or detectable issues observed with repeat dosing. However, there are two potential caveats associated with the antinociceptive results generated. The first is that antinociceptive efficacy was only detected with the use of a reflexive assay. This is a concern given the growing appreciation that changes in nociceptive reflex responses may not be reflective of changes in pain per se (King et al., 2009). However, this concern is appropriately focused on the potential impact of test compounds on spinal cord circuitry, rather than afferent activity, per se. And while it is possible that the attenuation of cold hypersensitivity observed is due to an action of VBJ103 on TRPM8 channels present somewhere other than the peripheral terminals of cold responsive afferents, we suggest these are the most likely target mediating the effects observed and consequently, likely to attenuate both withdrawal latency and the perception of cold pain. The second caveat is a potential impact of VBJ103 on motor responses, which could have also influenced the withdrawal latency. And while we did not directly assess the impact of VBJ103 on either gross- or fine-motor performance, there were no obvious changes in coordination detected by the investigator performing the nociceptive testing. Similarly, no changes in activity were detected even in response to VBJ103 doses an order of magnitude higher than those used in the nociceptive assays. Thus, we suggest systemic administration of VBJ103 is likely to block cold pain in patients suffering from chemotherapeutic-induced peripheral neuropathy.

Our nociceptive test yielded two potentially surprising results. The first is the absence of a detectable influence of VBJ103 on the response to noxious cold stimuli in oxaliplatin vehicle treated mice. One possible explanation is that VBJ103 is only blocking the increase in TRPM8 channels thought to mediate chemotherapy-induced cold hypersensitivity (Gauchan et al., 2009). It is also possible that other channels contribute to the baseline response to noxious cold stimuli (Luiz et al., 2019). The second surprising result was that HC-030031 had no detectable influence on oxaliplatin-induced cold hypersensitivity. While this is consistent with several previous reports (Descoeur et al., 2011; Zygmunt et al., 2022), it is in contrast with others (Materazzi et al., 2012). Aside from potential differences associated with the chemotherapeutic used, we do not have a good explanation for these discrepant results, except to again raise the possibility that other channels may contribute to cold sensitivity.

The most striking observation of the study is the impact of route of administration on the changes in CBT associated with TRPM8 antagonist administration. There are several mechanisms that may account for the differential influence of route of administration. Gut and liver metabolism of the test agents delivered i.p. is unlikely to account for the route of administration effect given that the influence of PBMC on CBT was lost in TRPM8 knock-out mice (Knowlton et al., 2011), and the fact that comparable results were obtained with PBMC and VBJ103, two very different molecules. Differential distribution of TRPM8 receptors may be involved in regulation of CBT. There is some evidence of a role for TRPM8 on CNS targets in the regulation of CBT (Reimundez et al., 2022). If such a mechanism contributed to the route of administration effect one might have predicted to see a dose by time interaction given that VBJ103 gets into the CNS with s.c. administration. However, our i.c.v. administration results argue against differential access to targets in the brain. Another possibility is the expression of TRPM8 on afferents innervating visceral structures, where a drop in core body temperature would have far greater influence on survival than a drop in temperature in the peripheral appendages. In this regard, while there does not appear to be TRPM8 expression in nodose ganglion neurons giving rise to vagal afferents, it is detectable on afferents innervating visceral structures (Hockley et al., 2019; Meerschaert et al., 2020). Nevertheless, given that both routes of administration appear to result in a systemic distribution of the antagonists, additional pharmacokinetic studies will be needed to see if a differential distribution of drug could account for a differential distribution of receptors on the changes in core body temperature.

In conclusion, we have provided evidence that it is possible to mitigate the impact of TRPM8 antagonists on CBT while retaining therapeutic efficacy via a subcutaneous route of administration. Additional experiments will be needed to determine the basis for this effect. Nevertheless, there remain no consistently effective treatments for cold hypersensitivity associated with the administration of chemotherapeutics, a side effect that may be dose limiting for these life-saving medications (Argyriou et al., 2008; Attal et al., 2009). Our results suggest that it may be possible to mitigate a major side effect of TRPM8 antagonists via route of administration, which may have a significant clinical impact on those undergoing treatment, as well as cancer survivors.

## Supplementary Material

Supporting information

## Data Availability

The data that support the findings of this study are available from the corresponding author upon reasonable request. A CCDC Deposition Number 2282159 contain the supplementary crystallographic data. Upon publication, this data can be obtained from the joint CCDC/FIZ Karlsruhe deposition service (https://www.ccdc.cam.ac.uk/structures/) and the Cambridge Structural Database. In addition, these data are supplied in the supporting information.

## What is already known

- Previously reported TRPM8 antagonists, while able to effectively relieve cold hypersensitivity, dysregulate core body temperature.

## What does this study add

- VBJ103 attenuates cold hypersensitivity with a minimal drop in core body temperature.
- This latter action depends on the route of administration.

## What is the clinical significance

- TRPM8 is a promising target for treating cold hypersensitivity associated with peripheral neuropathy.

## Abbreviations:

AAALAC: Association for Assessment and Accreditation of Laboratory Animal Care International
ACN: acetonitrile
AITC: allyl isothiocyanate
AUC: area under curve
CBT: core body temperature
DCM: dichloromethane
EDCI: N-ethyl-N′-(3-dimethylaminopropyl)carbodiimide hydrochloride
FOB: fold over baseline
HEPA: high-efficiency particulate air
HOBt: 1-hydroxybenzotriazole hydrate
PMT: photomultiplier tube
QC: quality controls
RM: repeated measures
TFR: transfection reagent
THF: tetrahydrofuran
