# Supplementary material for: Subcutaneous administration of a novel TRPM8 antagonist reverses cold hypersensitivity while attenuating the drop in core body temperature
Source: Br J Pharmacol. Author manuscript; Available in PMC 2025 Sep 24. (PMC12459314; doi:10.1111/bph.16429)
Supplement: Supporting information [file NIHMS2111829-supplement-Supporting_information.pdf]

Supplementary Information

Title: Subcutaneous administration of a novel TRPM8 antagonist reverses cold hypersensitivity while attenuating the drop in core body temperature

Michael S. Gold, Jorge B. Pineda-Farias, David Close, Smith Patel, Paul A. Johnston, Sean D. Stocker, V. Blair Journigan

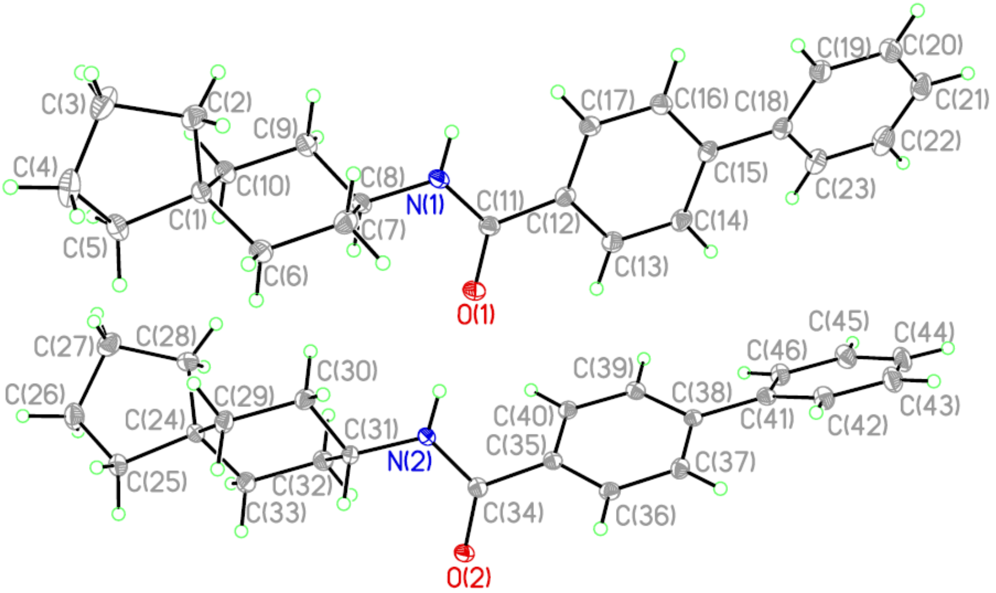

Figure S1. X-ray crystal structure of VBJ103. There are two molecules of VBJ103 which differ only in the orientation of the rings. There is also a solvate molecule of dioxane present.

Table S1. Sample and crystal data for VBJ103.

**Identification code**	VBJ103

**Chemical formula**	$C_{25}H_{31}NO_2$	
**Formula weight**	377.51 g/mol	
**Temperature**	100(2) K	
**Wavelength**	1.54178 Å	
**Crystal size**	0.030 x 0.060 x 0.180 mm	
**Crystal habit**	clear colourless blade	
**Crystal system**	triclinic	
**Space group**	P -1	
**Unit cell dimensions**	a = 9.7935(3) Å	α = 80.120(2)°
	b = 10.0923(3) Å	β = 84.886(2)°
	c = 24.2140(7) Å	γ = 61.7040(10)°
**Volume**	2075.98(11) $Å^3$	
**Z**	4	
**Density (calculated)**	1.208 g/cm^3	
**Absorption coefficient**	0.587 mm^{-1}	
**F(000)**	816	

Table S2. Data collection and structure refinement for VBJ103.

**Diffractometer**	Bruker Smart Apex II CCD
**Radiation source**	Bruker X8 Prospector Ultra IMuS (CuKα, λ = 1.54178 Å)
**Theta range for data collection**	1.85 to 68.30°
**Reflections collected**	45097
**Independent reflections**	7428 [R(int) = 0.0327]
**Coverage of independent reflections**	97.5%
**Absorption correction**	Multi-Scan
**Max. and min. transmission**	0.7500 and 0.6500
**Structure solution technique**	direct methods
**Structure solution program**	SHELXT 2014/5 (Sheldrick, 2014)
**Refinement method**	Full-matrix least-squares on F^2
**Refinement program**	SHELXL-2017/1 (Sheldrick, 2017)
**Function minimized**	$\Sigma w(F_o^2 - F_c^2)^2$
**Data / restraints / parameters**	7428 / 0 / 506

**Goodness-of-fit on F^2**	1.008	
**Final R indices**	6749 data; I>2σ(I)	R1 = 0.0321, wR2 = 0.0875
	all data	R1 = 0.0371, wR2 = 0.0901
**Weighting scheme**	$w=1/[\sigma^2(F_o^2)+(0.0680P)^2]$ where $P=(F_o^2+2F_c^2)/3$	
**Largest diff. peak and hole**	0.138 and -0.183 eÅ^{-3}	
**R.M.S. deviation from mean**	0.038 eÅ^{-3}	

Table S3. Atomic coordinates and equivalent isotropic atomic displacement parameters (Å^2) for VBJ103.

U(eq) is defined as one third of the trace of the orthogonalized U_{ij} tensor.

	**x/a**	**y/b**	**z/c**	**U(eq)**
O1	0.68286(13)	0.58606(11)	0.31596(4)	0.0271(2)
O2	0.71705(11)	0.06594(11)	0.29545(4)	0.0212(2)
O3	0.94074(14)	0.89047(12)	0.14836(5)	0.0390(3)
O4	0.14766(13)	0.60775(13)	0.20722(5)	0.0391(3)
N1	0.69384(14)	0.37718(13)	0.28964(5)	0.0230(3)
N2	0.62021(14)	0.91096(13)	0.28050(5)	0.0193(2)
C1	0.70092(17)	0.42385(16)	0.10771(6)	0.0219(3)
C2	0.76955(19)	0.25475(18)	0.10015(6)	0.0284(3)
C3	0.7489(2)	0.2544(2)	0.03850(7)	0.0392(4)
C4	0.7647(2)	0.3913(2)	0.00875(7)	0.0378(4)
C5	0.6749(2)	0.51074(19)	0.04684(6)	0.0311(4)
C6	0.81505(18)	0.44965(18)	0.13832(6)	0.0255(3)
C7	0.83549(17)	0.37679(18)	0.19995(6)	0.0259(3)
C8	0.67889(17)	0.43971(16)	0.23004(6)	0.0215(3)
C9	0.56396(17)	0.41039(17)	0.20222(6)	0.0232(3)
C10	0.54715(17)	0.48035(16)	0.14016(6)	0.0232(3)
C11	0.69494(16)	0.45669(16)	0.32856(6)	0.0201(3)
C12	0.71154(16)	0.38308(16)	0.38862(6)	0.0189(3)
C13	0.69793(17)	0.47134(17)	0.42907(6)	0.0246(3)
C14	0.71168(17)	0.41321(17)	0.48555(6)	0.0244(3)
C15	0.74077(15)	0.26371(16)	0.50380(6)	0.0197(3)

	x/a	y/b	z/c	U(eq)
C16	0.75308(17)	0.17605(17)	0.46294(6)	0.0230(3)
C17	0.73904(17)	0.23436(16)	0.40621(6)	0.0220(3)
C18	0.75934(15)	0.19866(17)	0.56437(6)	0.0202(3)
C19	0.84091(17)	0.04155(18)	0.58143(6)	0.0253(3)
C20	0.86019(19)	0.9811(2)	0.63804(7)	0.0322(4)
C21	0.79866(19)	0.0763(2)	0.67836(7)	0.0338(4)
C22	0.71606(19)	0.2320(2)	0.66219(7)	0.0335(4)
C23	0.69688(18)	0.29304(19)	0.60592(6)	0.0277(3)
C24	0.40081(16)	0.07152(16)	0.11632(6)	0.0202(3)
C25	0.38244(18)	0.15113(18)	0.05440(6)	0.0271(3)
C26	0.23469(19)	0.1633(2)	0.03281(6)	0.0315(4)
C27	0.22704(18)	0.02436(19)	0.06553(7)	0.0302(4)
C28	0.27281(17)	0.02126(17)	0.12448(6)	0.0241(3)
C29	0.56249(16)	0.93397(17)	0.12545(6)	0.0221(3)
C30	0.59705(16)	0.86184(16)	0.18671(6)	0.0207(3)
C31	0.57852(16)	0.98002(15)	0.22240(5)	0.0186(3)
C32	0.41361(17)	0.11114(16)	0.21742(6)	0.0216(3)
C33	0.37865(17)	0.18368(16)	0.15627(6)	0.0231(3)
C34	0.68268(15)	0.96268(15)	0.31309(6)	0.0178(3)
C35	0.70687(16)	0.88975(15)	0.37311(5)	0.0168(3)
C36	0.84654(16)	0.84538(16)	0.39920(6)	0.0189(3)
C37	0.86621(16)	0.78453(15)	0.45561(6)	0.0195(3)
C38	0.74647(16)	0.77100(14)	0.48769(5)	0.0165(3)
C39	0.60660(16)	0.81769(15)	0.46098(6)	0.0181(3)
C40	0.58782(16)	0.87407(15)	0.40433(6)	0.0179(3)
C41	0.76584(16)	0.71029(15)	0.54869(6)	0.0191(3)
C42	0.91181(17)	0.60896(17)	0.57135(6)	0.0245(3)
C43	0.92940(19)	0.55646(18)	0.62842(6)	0.0292(3)
C44	0.80177(19)	0.60280(18)	0.66392(6)	0.0288(3)
C45	0.65577(19)	0.70016(17)	0.64191(6)	0.0275(3)
C46	0.63779(17)	0.75409(16)	0.58500(6)	0.0228(3)
C47	0.0876(2)	0.61891(18)	0.15449(7)	0.0333(4)
C48	0.0501(2)	0.76926(18)	0.11998(7)	0.0352(4)
C49	0.9998(2)	0.87929(19)	0.20149(7)	0.0335(4)
C50	0.03681(19)	0.72819(19)	0.23587(7)	0.0318(4)

Table S4. Bond lengths (Å) for VBJ103.

O1-C11	1.2390(17)	O2-C34	1.2394(17)
O3-C48	1.4251(19)	O3-C49	1.423(2)
O4-C47	1.418(2)	O4-C50	1.429(2)
N1-C11	1.3424(18)	N1-C8	1.4628(18)
N1-H1	0.880000	N2-C34	1.3416(18)
N2-C31	1.4587(17)	N2-H2	0.880000
C1-C6	1.535(2)	C1-C10	1.529(2)
C1-C2	1.548(2)	C1-C5	1.555(2)
C2-C3	1.525(2)	C2-H2A	0.990000
C2-H2B	0.990000	C3-C4	1.515(3)
C3-H3A	0.990000	C3-H3B	0.990000
C4-C5	1.519(2)	C4-H4A	0.990000
C4-H4B	0.990000	C5-H5A	0.990000
C5-H5B	0.990000	C6-C7	1.531(2)
C6-H6A	0.990000	C6-H6B	0.990000
C7-C8	1.524(2)	C7-H7A	0.990000
C7-H7B	0.990000	C8-C9	1.526(2)
C8-H8	1.000000	C9-C10	1.534(2)
C9-H9A	0.990000	C9-H9B	0.990000
C10-H10A	0.990000	C10-H10B	0.990000
C11-C12	1.500(2)	C12-C17	1.387(2)
C12-C13	1.391(2)	C13-C14	1.384(2)
C13-H13	0.950000	C14-C15	1.392(2)
C14-H14	0.950000	C15-C16	1.398(2)
C15-C18	1.490(2)	C16-C17	1.390(2)
C16-H16	0.950000	C17-H17	0.950000
C18-C19	1.399(2)	C18-C23	1.400(2)
C19-C20	1.392(2)	C19-H19	0.950000
C20-C21	1.381(2)	C20-H20	0.950000
C21-C22	1.384(2)	C21-H21	0.950000
C22-C23	1.385(2)	C22-H22	0.950000
C23-H23	0.950000	C24-C29	1.535(2)
C24-C33	1.539(2)	C24-C28	1.5466(19)
C24-C25	1.5568(19)	C25-C26	1.526(2)
C25-H25A	0.990000	C25-H25B	0.990000
C26-C27	1.519(2)	C26-H26A	0.990000
C26-H26B	0.990000	C27-C28	1.525(2)

C27-H27A	0.990000	C27-H27B	0.990000
C28-H28A	0.990000	C28-H28B	0.990000
C29-C30	1.5264(19)	C29-H29A	0.990000
C29-H29B	0.990000	C30-C31	1.5242(18)
C30-H30A	0.990000	C30-H30B	0.990000
C31-C32	1.5257(19)	C31-H31	1.000000
C32-C33	1.5253(19)	C32-H32A	0.990000
C32-H32B	0.990000	C33-H33A	0.990000
C33-H33B	0.990000	C34-C35	1.4962(19)
C35-C40	1.3902(19)	C35-C36	1.3953(19)
C36-C37	1.388(2)	C36-H36	0.950000
C37-C38	1.3965(19)	C37-H37	0.950000
C38-C39	1.3994(19)	C38-C41	1.4908(19)
C39-C40	1.3844(19)	C39-H39	0.950000
C40-H40	0.950000	C41-C46	1.397(2)
C41-C42	1.397(2)	C42-C43	1.387(2)
C42-H42	0.950000	C43-C44	1.383(2)
C43-H43	0.950000	C44-C45	1.385(2)
C44-H44	0.950000	C45-C46	1.386(2)
C45-H45	0.950000	C46-H46	0.950000
C47-C48	1.492(2)	C47-H47A	0.990000
C47-H47B	0.990000	C48-H48A	0.990000
C48-H48B	0.990000	C49-C50	1.498(2)
C49-H49A	0.990000	C49-H49B	0.990000
C50-H50A	0.990000	C50-H50B	0.990000

Table S5. Bond angles (°) for VBJ103.

C48-O3-C49	109.84(12)	C47-O4-C50	109.40(12)
C11-N1-C8	121.23(12)	C11-N1-H1	119.400000
C8-N1-H1	119.400000	C34-N2-C31	121.83(11)
C34-N2-H2	119.100000	C31-N2-H2	119.100000
C6-C1-C10	108.61(12)	C6-C1-C2	110.94(12)
C10-C1-C2	112.49(12)	C6-C1-C5	110.44(12)
C10-C1-C5	110.06(12)	C2-C1-C5	104.26(12)
C3-C2-C1	106.16(12)	C3-C2-H2A	110.500000
C1-C2-H2A	110.500000	C3-C2-H2B	110.500000
C1-C2-H2B	110.500000	H2A-C2-H2B	108.700000

C4-C3-C2	103.67(13)	C4-C3-H3A	111.000000
C2-C3-H3A	111.000000	C4-C3-H3B	111.000000
C2-C3-H3B	111.000000	H3A-C3-H3B	109.000000
C3-C4-C5	102.97(14)	C3-C4-H4A	111.200000
C5-C4-H4A	111.200000	C3-C4-H4B	111.200000
C5-C4-H4B	111.200000	H4A-C4-H4B	109.100000
C4-C5-C1	106.25(13)	C4-C5-H5A	110.500000
C1-C5-H5A	110.500000	C4-C5-H5B	110.500000
C1-C5-H5B	110.500000	H5A-C5-H5B	108.700000
C1-C6-C7	112.75(12)	C1-C6-H6A	109.000000
C7-C6-H6A	109.000000	C1-C6-H6B	109.000000
C7-C6-H6B	109.000000	H6A-C6-H6B	107.800000
C8-C7-C6	109.73(12)	C8-C7-H7A	109.700000
C6-C7-H7A	109.700000	C8-C7-H7B	109.700000
C6-C7-H7B	109.700000	H7A-C7-H7B	108.200000
N1-C8-C9	110.68(11)	N1-C8-C7	111.57(12)
C9-C8-C7	110.99(12)	N1-C8-H8	107.800000
C9-C8-H8	107.800000	C7-C8-H8	107.800000
C8-C9-C10	109.73(12)	C8-C9-H9A	109.700000
C10-C9-H9A	109.700000	C8-C9-H9B	109.700000
C10-C9-H9B	109.700000	H9A-C9-H9B	108.200000
C9-C10-C1	113.63(12)	C9-C10-H10A	108.800000
C1-C10-H10A	108.800000	C9-C10-H10B	108.800000
C1-C10-H10B	108.800000	H10A-C10-H10B	107.700000
O1-C11-N1	121.92(13)	O1-C11-C12	120.58(13)
N1-C11-C12	117.49(13)	C17-C12-C13	118.33(13)
C17-C12-C11	124.55(13)	C13-C12-C11	117.12(13)
C14-C13-C12	121.26(14)	C14-C13-H13	119.400000
C12-C13-H13	119.400000	C13-C14-C15	121.04(13)
C13-C14-H14	119.500000	C15-C14-H14	119.500000
C14-C15-C16	117.38(13)	C14-C15-C18	121.59(13)
C16-C15-C18	121.03(14)	C17-C16-C15	121.63(14)
C17-C16-H16	119.200000	C15-C16-H16	119.200000
C16-C17-C12	120.34(14)	C16-C17-H17	119.800000
C12-C17-H17	119.800000	C19-C18-C23	118.01(14)
C19-C18-C15	120.98(14)	C23-C18-C15	121.00(14)
C20-C19-C18	120.92(15)	C20-C19-H19	119.500000

C18-C19-H19	119.500000	C21-C20-C19	120.13(16)
C21-C20-H20	119.900000	C19-C20-H20	119.900000
C20-C21-C22	119.69(15)	C20-C21-H21	120.200000
C22-C21-H21	120.200000	C23-C22-C21	120.51(16)
C23-C22-H22	119.700000	C21-C22-H22	119.700000
C22-C23-C18	120.73(16)	C22-C23-H23	119.600000
C18-C23-H23	119.600000	C29-C24-C33	108.86(11)
C29-C24-C28	111.14(11)	C33-C24-C28	111.93(12)
C29-C24-C25	110.52(12)	C33-C24-C25	110.14(12)
C28-C24-C25	104.20(11)	C26-C25-C24	106.62(12)
C26-C25-H25A	110.400000	C24-C25-H25A	110.400000
C26-C25-H25B	110.400000	C24-C25-H25B	110.400000
H25A-C25-H25B	108.600000	C25-C26-C27	103.06(12)
C25-C26-H26A	111.200000	C27-C26-H26A	111.200000
C25-C26-H26B	111.200000	C27-C26-H26B	111.200000
H26A-C26-H26B	109.100000	C28-C27-C26	102.95(12)
C28-C27-H27A	111.200000	C26-C27-H27A	111.200000
C28-C27-H27B	111.200000	C26-C27-H27B	111.200000
H27A-C27-H27B	109.100000	C27-C28-C24	105.49(11)
C27-C28-H28A	110.600000	C24-C28-H28A	110.600000
C27-C28-H28B	110.600000	C24-C28-H28B	110.600000
H28A-C28-H28B	108.800000	C24-C29-C30	113.66(11)
C24-C29-H29A	108.800000	C30-C29-H29A	108.800000
C24-C29-H29B	108.800000	C30-C29-H29B	108.800000
H29A-C29-H29B	107.700000	C31-C30-C29	110.22(11)

C31-C30-H30A	109.600000	C29-C30-H30A	109.600000
C31-C30-H30B	109.600000	C29-C30-H30B	109.600000
H30A-C30-H30B	108.100000	N2-C31-C30	110.94(11)
N2-C31-C32	112.03(11)	C30-C31-C32	109.84(11)
N2-C31-H31	108.000000	C30-C31-H31	108.000000
C32-C31-H31	108.000000	C31-C32-C33	109.86(12)
C31-C32-H32A	109.700000	C33-C32-H32A	109.700000
C31-C32-H32B	109.700000	C33-C32-H32B	109.700000
H32A-C32-H32B	108.200000	C32-C33-C24	114.01(12)
C32-C33-H33A	108.800000	C24-C33-H33A	108.800000
C32-C33-H33B	108.800000	C24-C33-H33B	108.800000
H33A-C33-H33B	107.600000	O2-C34-N2	122.84(12)
O2-C34-C35	121.18(12)	N2-C34-C35	115.97(12)
C40-C35-C36	119.32(12)	C40-C35-C34	120.58(12)
C36-C35-C34	119.98(12)	C37-C36-C35	119.99(13)
C37-C36-H36	120.000000	C35-C36-H36	120.000000
C36-C37-C38	121.21(13)	C36-C37-H37	119.400000
C38-C37-H37	119.400000	C37-C38-C39	117.98(12)
C37-C38-C41	121.23(12)	C39-C38-C41	120.79(12)
C40-C39-C38	121.10(13)	C40-C39-H39	119.400000
C38-C39-H39	119.400000	C39-C40-C35	120.34(13)
C39-C40-H40	119.800000	C35-C40-H40	119.800000
C46-C41-C42	118.03(13)	C46-C41-C38	120.69(13)
C42-C41-C38	121.28(13)	C43-C42-C41	120.91(14)
C43-C42-H42	119.500000	C41-C42-H42	119.500000
C44-C43-C42	120.33(15)	C44-C43-H43	119.800000
C42-C43-H43	119.800000	C43-C44-C45	119.45(14)
C43-C44-H44	120.300000	C45-C44-H44	120.300000
C46-C45-C44	120.41(15)	C46-C45-H45	119.800000
C44-C45-H45	119.800000	C45-C46-C41	120.83(14)
C45-C46-H46	119.600000	C41-C46-H46	119.600000

O4-C47-C48	110.70(14)	O4-C47-H47A	109.500000
C48-C47-H47A	109.500000	O4-C47-H47B	109.500000
C48-C47-H47B	109.500000	H47A-C47-H47B	108.100000
O3-C48-C47	110.72(14)	O3-C48-H48A	109.500000
C47-C48-H48A	109.500000	O3-C48-H48B	109.500000
C47-C48-H48B	109.500000	H48A-C48-H48B	108.100000
O3-C49-C50	110.51(13)	O3-C49-H49A	109.500000
C50-C49-H49A	109.500000	O3-C49-H49B	109.500000
C50-C49-H49B	109.500000	H49A-C49-H49B	108.100000
O4-C50-C49	110.28(13)	O4-C50-H50A	109.600000
C49-C50-H50A	109.600000	O4-C50-H50B	109.600000
C49-C50-H50B	109.600000	H50A-C50-H50B	108.100000

Table S6. Anisotropic atomic displacement parameters (Å^2) for VBJ103.

The anisotropic atomic displacement factor exponent takes the form: $-2\pi^2[h^2 a^{*2} U_{11} + ... + 2 h k a^* b^* U_{12}]$

	U_{11}	U_{22}	U_{33}	U_{23}	U_{13}	U_{12}
O1	0.0411(6)	0.0160(5)	0.0259(6)	-0.0007(4)	-0.0057(4)	-0.0147(4)
O2	0.0300(5)	0.0193(5)	0.0186(5)	-0.0020(4)	0.0003(4)	-0.0155(4)
O3	0.0441(7)	0.0223(6)	0.0319(6)	-0.0016(5)	0.0006(5)	-0.0014(5)
O4	0.0306(6)	0.0317(7)	0.0356(7)	0.0045(5)	-0.0016(5)	-0.0017(5)
N1	0.0367(7)	0.0175(6)	0.0178(6)	-0.0004(5)	-0.0011(5)	-0.0157(5)
N2	0.0278(6)	0.0182(6)	0.0147(6)	0.0002(4)	-0.0017(5)	-0.0138(5)
C1	0.0283(8)	0.0200(8)	0.0169(7)	-0.0005(5)	-0.0017(6)	-0.0114(6)

	U_{11}	U_{22}	U_{33}	U_{23}	U_{13}	U_{12}
C2	0.0335(8)	0.0236(8)	0.0275(8)	-0.0069(6)	-0.0021(6)	-0.0116(7)
C3	0.0422(10)	0.0369(10)	0.0357(10)	-0.0159(8)	-0.0105(8)	-0.0112(8)
C4	0.0325(9)	0.0554(12)	0.0215(8)	-0.0096(7)	-0.0023(7)	-0.0158(8)
C5	0.0426(9)	0.0299(9)	0.0206(8)	0.0025(6)	-0.0023(7)	-0.0185(7)
C6	0.0297(8)	0.0300(9)	0.0217(8)	-0.0040(6)	0.0037(6)	-0.0184(7)
C7	0.0267(8)	0.0309(9)	0.0248(8)	-0.0037(6)	-0.0020(6)	-0.0170(7)
C8	0.0302(8)	0.0179(7)	0.0170(7)	-0.0011(5)	0.0001(6)	-0.0122(6)
C9	0.0234(7)	0.0246(8)	0.0204(7)	0.0000(6)	-0.0007(6)	-0.0111(6)
C10	0.0242(7)	0.0195(8)	0.0225(8)	0.0012(6)	-0.0041(6)	-0.0082(6)
C11	0.0204(7)	0.0172(7)	0.0227(7)	-0.0019(5)	-0.0013(6)	-0.0090(6)
C12	0.0174(7)	0.0181(7)	0.0216(7)	-0.0042(5)	-0.0013(5)	-0.0079(6)
C13	0.0304(8)	0.0172(8)	0.0253(8)	-0.0036(6)	-0.0054(6)	-0.0094(6)
C14	0.0288(8)	0.0208(8)	0.0234(8)	-0.0082(6)	-0.0028(6)	-0.0094(6)
C15	0.0146(7)	0.0242(8)	0.0213(7)	-0.0050(6)	-0.0009(5)	-0.0091(6)
C16	0.0273(8)	0.0211(8)	0.0221(7)	-0.0034(6)	0.0004(6)	-0.0125(6)
C17	0.0279(8)	0.0200(8)	0.0203(7)	-0.0047(6)	0.0005(6)	-0.0127(6)
C18	0.0144(7)	0.0281(8)	0.0205(7)	-0.0039(6)	0.0002(5)	-0.0119(6)
C19	0.0272(8)	0.0303(9)	0.0226(8)	-0.0023(6)	-0.0008(6)	-0.0173(7)
C20	0.0363(9)	0.0376(10)	0.0288(9)	0.0060(7)	-0.0064(7)	-0.0246(8)
C21	0.0369(9)	0.0542(12)	0.0201(8)	0.0014(7)	-0.0022(6)	-0.0310(9)

	U_{11}	U_{22}	U_{33}	U_{23}	U_{13}	U_{12}
C22	0.0291(8)	0.0549(12)	0.0214(8)	-0.0117(7)	0.0052(6)	-0.0225(8)
C23	0.0241(8)	0.0341(9)	0.0257(8)	-0.0078(6)	-0.0013(6)	-0.0130(7)
C24	0.0242(7)	0.0229(8)	0.0165(7)	0.0012(6)	-0.0022(5)	-0.0144(6)
C25	0.0327(8)	0.0328(9)	0.0199(8)	0.0031(6)	-0.0068(6)	-0.0198(7)
C26	0.0305(8)	0.0426(10)	0.0216(8)	0.0011(7)	-0.0058(6)	-0.0182(7)
C27	0.0267(8)	0.0387(10)	0.0318(9)	-0.0087(7)	-0.0007(7)	-0.0194(7)
C28	0.0255(7)	0.0256(8)	0.0255(8)	-0.0012(6)	-0.0016(6)	-0.0161(6)
C29	0.0236(7)	0.0264(8)	0.0178(7)	-0.0062(6)	0.0004(6)	-0.0121(6)
C30	0.0218(7)	0.0177(7)	0.0218(7)	-0.0042(5)	-0.0026(5)	-0.0077(6)
C31	0.0241(7)	0.0192(7)	0.0150(7)	-0.0013(5)	-0.0026(5)	-0.0121(6)
C32	0.0264(7)	0.0175(7)	0.0199(7)	-0.0036(5)	0.0002(6)	-0.0093(6)
C33	0.0276(8)	0.0180(8)	0.0225(7)	0.0003(6)	-0.0059(6)	-0.0098(6)
C34	0.0187(7)	0.0140(7)	0.0181(7)	-0.0035(5)	0.0006(5)	-0.0052(5)
C35	0.0209(7)	0.0110(7)	0.0174(7)	-0.0040(5)	-0.0001(5)	-0.0058(5)
C36	0.0221(7)	0.0185(7)	0.0190(7)	-0.0051(5)	0.0029(5)	-0.0117(6)
C37	0.0178(7)	0.0193(8)	0.0198(7)	-0.0027(5)	-0.0031(5)	-0.0069(6)
C38	0.0217(7)	0.0110(7)	0.0170(7)	-0.0041(5)	-0.0005(5)	-0.0072(5)
C39	0.0198(7)	0.0159(7)	0.0184(7)	-0.0039(5)	0.0025(5)	-0.0081(5)
C40	0.0196(7)	0.0152(7)	0.0189(7)	-0.0036(5)	-0.0014(5)	-0.0074(5)
C41	0.0255(7)	0.0139(7)	0.0186(7)	-0.0032(5)	-0.0011(6)	-0.0092(6)

	U11	U22	U33	U23	U13	U12
C42	0.0252(7)	0.0226(8)	0.0224(8)	-0.0025(6)	-0.0008(6)	-0.0088(6)
C43	0.0331(9)	0.0238(8)	0.0244(8)	0.0021(6)	-0.0078(6)	-0.0088(7)
C44	0.0432(9)	0.0261(8)	0.0146(7)	0.0001(6)	-0.0037(6)	-0.0145(7)
C45	0.0350(9)	0.0265(9)	0.0184(7)	-0.0051(6)	0.0041(6)	-0.0125(7)
C46	0.0250(8)	0.0201(8)	0.0202(7)	-0.0048(6)	-0.0006(6)	-0.0075(6)
C47	0.0371(9)	0.0229(9)	0.0377(9)	-0.0073(7)	0.0048(7)	-0.0121(7)
C48	0.0423(10)	0.0268(9)	0.0279(8)	-0.0069(7)	0.0036(7)	-0.0088(7)
C49	0.0404(9)	0.0334(9)	0.0332(9)	-0.0128(7)	0.0109(7)	-0.0217(8)
C50	0.0283(8)	0.0374(9)	0.0268(8)	-0.0031(7)	0.0000(7)	-0.0136(7)

Table S7. Hydrogen atomic coordinates and isotropic atomic displacement parameters (Å^2) for VBJ103.

	x/a	y/b	z/c	U(eq)
H1	0.7023	0.2857	0.3002	0.028000
H2	0.6039	0.8332	0.2946	0.023000
H2A	0.8808	0.1987	0.1101	0.034000
H2B	0.7139	0.2061	0.1244	0.034000
H3A	0.8300	0.1595	0.0257	0.047000
H3B	0.6457	0.2649	0.0320	0.047000
H4A	0.7187	0.4246	-0.0291	0.045000
H4B	0.8748	0.3690	0.0054	0.045000
H5A	0.5632	0.5644	0.0377	0.037000
H5B	0.7137	0.5865	0.0425	0.037000
H6A	0.7772	0.5604	0.1362	0.031000
H6B	0.9171	0.4067	0.1191	0.031000
H7A	0.8805	0.2650	0.2025	0.031000
H7B	0.9076	0.3989	0.2181	0.031000
H8	0.6368	0.5523	0.2270	0.026000

	x/a	y/b	z/c	U(eq)
H9A	0.4619	0.4559	0.2213	0.028000
H9B	0.6011	0.2995	0.2055	0.028000
H10A	0.4993	0.5924	0.1375	0.028000
H10B	0.4761	0.4564	0.1223	0.028000
H13	0.6788	0.5735	0.4177	0.030000
H14	0.7011	0.4763	0.5123	0.029000
H16	0.7715	0.0741	0.4742	0.028000
H17	0.7483	0.1721	0.3793	0.026000
H19	0.8838	-0.0248	0.5540	0.030000
H20	0.9158	-0.1259	0.6490	0.039000
H21	0.8130	0.0352	0.7170	0.041000
H22	0.6722	0.2974	0.6899	0.040000
H23	0.6407	0.4001	0.5954	0.033000
H25A	0.3740	1.2535	0.0524	0.033000
H25B	0.4731	1.0905	0.0316	0.033000
H26A	0.2418	1.1600	-0.0080	0.038000
H26B	0.1428	1.2583	0.0407	0.038000
H27A	0.1211	1.0355	0.0652	0.036000
H27B	0.3007	0.9307	0.0501	0.036000
H28A	0.3127	0.9175	0.1459	0.029000
H28B	0.1826	1.0921	0.1449	0.029000
H29A	0.6414	0.9665	0.1111	0.027000
H29B	0.5718	0.8562	0.1033	0.027000
H30A	0.5252	0.8198	0.2005	0.025000
H30B	0.7043	0.7772	0.1899	0.025000
H31	0.6511	1.0217	0.2073	0.022000
H32A	0.4022	1.1882	0.2403	0.026000
H32B	0.3390	1.0728	0.2317	0.026000
H33A	0.4471	1.2305	0.1437	0.028000
H33B	0.2702	1.2660	0.1537	0.028000
H36	0.9282	0.8568	0.3784	0.023000
H37	0.9629	0.7515	0.4727	0.023000
H39	0.5231	0.8106	0.4821	0.022000
H40	0.4931	0.9022	0.3867	0.022000
H42	1.0003	0.5755	0.5473	0.029000
H43	1.0296	0.4883	0.6432	0.035000
H44	0.8141	0.5681	0.7031	0.035000

	x/a	y/b	z/c	U(eq)
H45	0.5674	0.7302	0.6660	0.033000
H46	0.5371	0.8217	0.5705	0.027000
H47A	-0.0073	0.6065	0.1602	0.040000
H47B	0.1649	0.5364	0.1344	0.040000
H48A	0.1462	0.7791	0.1123	0.042000
H48B	0.0070	0.7755	0.0836	0.042000
H49A	-0.0779	0.9617	0.2215	0.040000
H49B	0.0948	0.8917	0.1961	0.040000
H50A	0.0790	0.7215	0.2725	0.038000
H50B	-0.0591	0.7180	0.2430	0.038000

Compound Name	Target Class	Assay Name	Mode	Assay Target	Result Type	RC50 (µM)
NECA	GPCR	Calcium Flux	Agonist	ADORA2A	EC50	0.0162
SCH 442416	GPCR	Calcium Flux	Antagonist	ADORA2A	IC50	0.07677
A 61603 Hydrobromide	GPCR	Calcium Flux	Agonist	ADRA1A	EC50	6e-05
Tamsulosin	GPCR	Calcium Flux	Antagonist	ADRA1A	IC50	0.00068
UK 14304	GPCR	cAMP	Agonist	ADRA2A	EC50	2e-05
Yohimbine	GPCR	cAMP	Antagonist	ADRA2A	IC50	0.01042
(-)-Isoproterenol	GPCR	cAMP	Agonist	ADRB1	EC50	0.00062
Betaxolol	GPCR	cAMP	Antagonist	ADRB1	IC50	0.00234
(-)-Isoproterenol	GPCR	cAMP	Agonist	ADRB2	EC50	0.00069
ICI 118,551 hydrochloride	GPCR	cAMP	Antagonist	ADRB2	IC50	0.00044
[Arg8]-Vasopressin	GPCR	Calcium Flux	Agonist	AVPR1A	EC50	0.00039
SR 49059	GPCR	Calcium Flux	Antagonist	AVPR1A	IC50	0.00195
(Tyr[SO3H]27)Cholecystokinin fragment 26-33 Amide	GPCR	Calcium Flux	Agonist	CCKAR	EC50	6e-05
SR 27897	GPCR	Calcium Flux	Antagonist	CCKAR	IC50	0.04939
Acetylcholine chloride	GPCR	Calcium Flux	Agonist	CHRM1	EC50	0.01447
Atropine	GPCR	Calcium Flux	Antagonist	CHRM1	IC50	0.00286
Acetylcholine chloride	GPCR	cAMP	Agonist	CHRM2	EC50	0.02906
Atropine	GPCR	cAMP	Antagonist	CHRM2	IC50	0.00141
Acetylcholine chloride	GPCR	Calcium Flux	Agonist	CHRM3	EC50	0.03479
Atropine	GPCR	Calcium Flux	Antagonist	CHRM3	IC50	0.00148
CP 55940	GPCR	cAMP	Agonist	CNR1	EC50	4e-05
AM 251	GPCR	cAMP	Antagonist	CNR1	IC50	0.00731
CP 55940	GPCR	cAMP	Agonist	CNR2	EC50	0.00014
SR 144528	GPCR	cAMP	Antagonist	CNR2	IC50	0.08089
Dopamine	GPCR	cAMP	Agonist	DRD1	EC50	0.09783
SCH 39166	GPCR	cAMP	Antagonist	DRD1	IC50	0.00023
Dopamine	GPCR	cAMP	Agonist	DRD2S	EC50	0.00382
Risperidone	GPCR	cAMP	Antagonist	DRD2S	IC50	0.0023
Endothelin 1	GPCR	Calcium Flux	Agonist	EDNRA	EC50	0.00235
BMS 182874	GPCR	Calcium Flux	Antagonist	EDNRA	IC50	0.49706
Histamine	GPCR	Calcium Flux	Agonist	HRH1	EC50	0.01804
Mepyramine	GPCR	Calcium Flux	Antagonist	HRH1	IC50	0.00376
Histamine	GPCR	cAMP	Agonist	HRH2	EC50	0.82788
Tiotidine	GPCR	cAMP	Antagonist	HRH2	IC50	0.07814
Serotonin Hydrochloride	GPCR	cAMP	Agonist	HTR1A	EC50	0.00494
Spiperone	GPCR	cAMP	Antagonist	HTR1A	IC50	0.05602
Serotonin Hydrochloride	GPCR	cAMP	Agonist	HTR1B	EC50	0.00014
SB 224289	GPCR	cAMP	Antagonist	HTR1B	IC50	0.01384
Serotonin Hydrochloride	GPCR	Calcium Flux	Agonist	HTR2A	EC50	0.0016
Altanserin	GPCR	Calcium Flux	Antagonist	HTR2A	IC50	0.00607

Table S8. Table of control values for 78 assays tested in the SAFETYscan E/IC_{50} ELECT panel.

Compound Name	Target Class	Assay Name	Mode	Assay Target	Result Type	RC50 (µM)
Serotonin Hydrochloride	GPCR	Calcium Flux	Agonist	HTR2B	EC50	0.00403
LY 272015	GPCR	Calcium Flux	Antagonist	HTR2B	IC50	0.00024
DADLE	GPCR	cAMP	Agonist	OPRD1	EC50	0.00013
Naltriben	GPCR	cAMP	Antagonist	OPRD1	IC50	0.00023
Dynorphin A (1-17)	GPCR	cAMP	Agonist	OPRK1	EC50	0.00396
nor-Binaltorphimine	GPCR	cAMP	Antagonist	OPRK1	IC50	0.01136
DAMGO	GPCR	cAMP	Agonist	OPRM1	EC50	0.00249
Naloxone	GPCR	cAMP	Antagonist	OPRM1	IC50	0.00312
Isradipine	Ion Channel	Ion Channel	Blocker	CAV1.2	IC50	0.01256
Picrotoxin	Ion Channel	Ion Channel	Blocker	GABAA	IC50	2.69929
GABA	Ion Channel	Ion Channel	Opener	GABAA	EC50	4.08731
Astemizole	Ion Channel	Ion Channel	Blocker	hERG	IC50	0.37608
Bemesetron	Ion Channel	Ion Channel	Blocker	HTR3A	IC50	0.00139
Serotonin Hydrochloride	Ion Channel	Ion Channel	Opener	HTR3A	EC50	0.27666
XE 991	Ion Channel	Ion Channel	Blocker	KvLQT1/minK	IC50	1.26982
ML-277	Ion Channel	Ion Channel	Opener	KvLQT1/minK	EC50	3.432
Dihydro-ÃŸ-erythroidine	Ion Channel	Ion Channel	Blocker	nAChR(a4/b2)	IC50	0.58602
(-)-Nicotine	Ion Channel	Ion Channel	Opener	nAChR(a4/b2)	EC50	2.94853
Lidocaine	Ion Channel	Ion Channel	Blocker	NAV1.5	IC50	41.69009
(+)-MK 801 maleate	Ion Channel	Ion Channel	Blocker	NMDAR (1A/2B)	IC50	0.05583
L-Glutamic Acid	Ion Channel	Ion Channel	Opener	NMDAR (1A/2B)	EC50	0.35712
BMS-754807	Kinases	Binding	Inhibitor	INSR	IC50	0.00065
Gleevec	Kinases	Binding	Inhibitor	LCK	IC50	0.06463
Staurosporine	Kinases	Binding	Inhibitor	ROCK1	IC50	20.00011
SU-11248	Kinases	Binding	Inhibitor	VEGFR2	IC50	0.00032
BMS-564929	NHR	NHR Nuclear Translocation	Agonist	AR	EC50	0.00303
Geldanamycin	NHR	NHR Nuclear Translocation	Antagonist	AR	IC50	0.04329
Dexamethasone	NHR	NHR Protein Interaction	Agonist	GR	EC50	0.1148
Mifepristone	NHR	NHR Protein Interaction	Antagonist	GR	IC50	0.03097
Physostigmine	Non-Kinase Enzymes	Enzymatic	Inhibitor	AChE	IC50	0.02652
Indomethacin	Non-Kinase Enzymes	Enzymatic	Inhibitor	COX1	IC50	0.02965
NS-398	Non-Kinase Enzymes	Enzymatic	Inhibitor	COX2	IC50	0.03533
Clorgyline	Non-Kinase Enzymes	Enzymatic	Inhibitor	MAOA	IC50	0.00126
Cilostamide	Non-Kinase Enzymes	Enzymatic	Inhibitor	PDE3A	IC50	0.04227
Cilomilast	Non-Kinase Enzymes	Enzymatic	Inhibitor	PDE4D2	IC50	0.01312
GBR 12909	Transporter	Transporter	Blocker	DAT	IC50	0.00547
Desipramine	Transporter	Transporter	Blocker	NET	IC50	0.02529
Clomipramine	Transporter	Transporter	Blocker	SERT	IC50	0.01203

Table S8 (contd).

VBJ103
ADORA2A Agonist
RC50 (uM) > 100

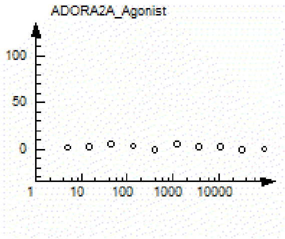

VBJ103
ADORA2A Antagonist
RC50 (uM) > 100

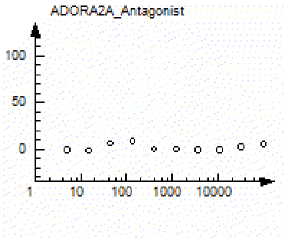

VBJ103
ADRA1A Agonist
RC50 (uM) > 100

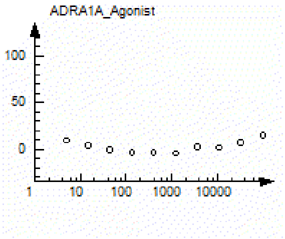

VBJ103
ADRA1A Antagonist
RC50 (uM) > 100

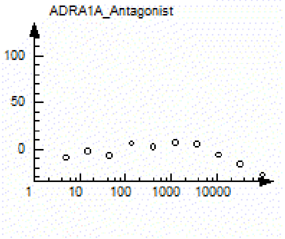

VBJ103
ADRA2A Agonist
RC50 (uM) > 100

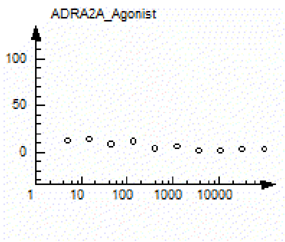

VBJ103
ADRA2A Antagonist
RC50 (uM) > 100

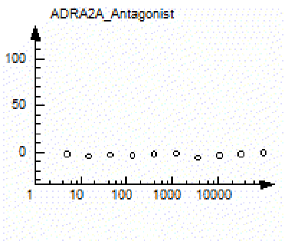

VBJ103
ADRB1 Agonist
RC50 (uM) > 100

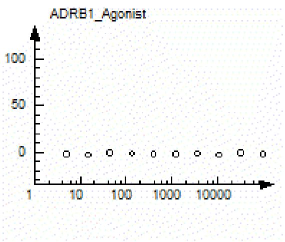

VBJ103
ADRB1 Antagonist
RC50 (uM) > 100

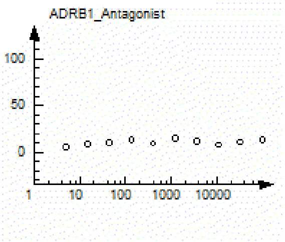

VBJ103
ADRB2 Agonist
RC50 (uM) > 100

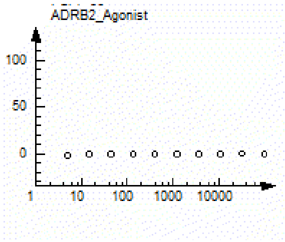

VBJ103
ADRB2 Antagonist
RC50 (uM) > 100

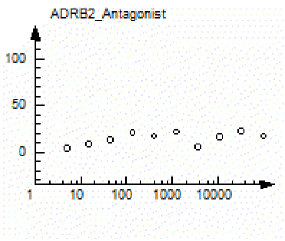

VBJ103
AVPR1A Agonist
RC50 (uM) > 100

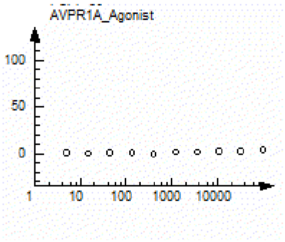

VBJ103
AVPR1A Antagonist
RC50 (uM) > 100

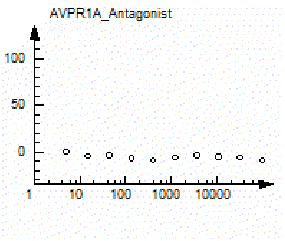

VBJ103
CCKAR Agonist
RC50 (uM) > 100

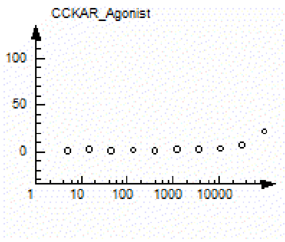

VBJ103
CCKAR Antagonist
RC50 (uM) > 100

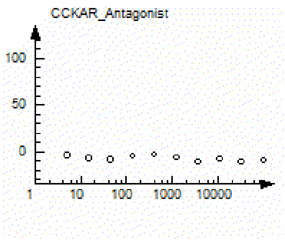

VBJ103
CHRM1 Agonist
RC50 (uM) > 100

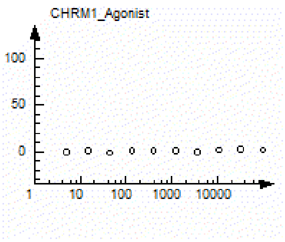

VBJ103
CHRM1 Antagonist
RC50 (uM) > 100

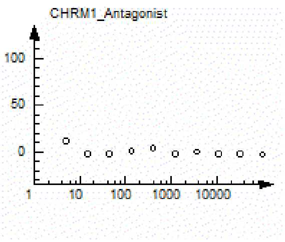

VBJ103
CHRM2 Agonist
RC50 (uM) > 100

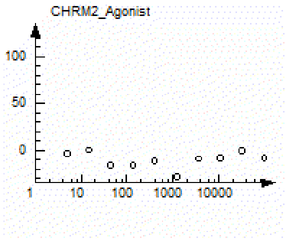

VBJ103
CHRM2 Antagonist
RC50 (uM) > 100

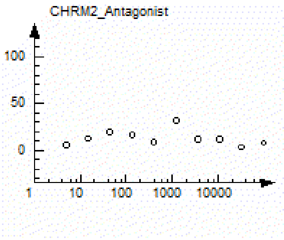

VBJ103
CHRM3 Agonist
RC50 (uM) > 100

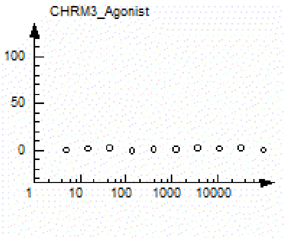

VBJ103
CHRM3 Antagonist
RC50 (uM) > 100

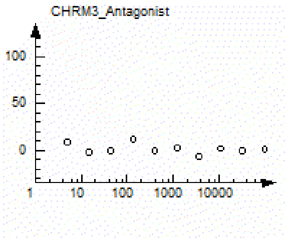

VBJ103
CNR1 Agonist
RC50 (uM) > 100

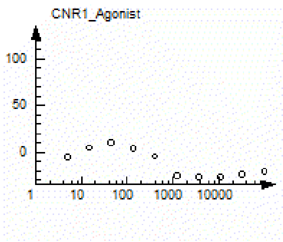

VBJ103
CNR1 Antagonist
RC50 (uM) > 100

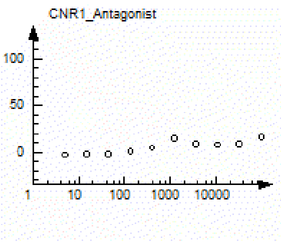

VBJ103
CNR2 Agonist
RC50 (uM) > 100

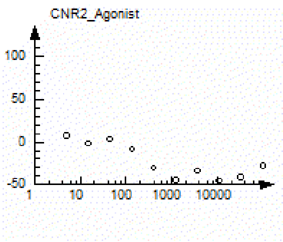

VBJ103
CNR2 Antagonist
RC50 (uM) > 100

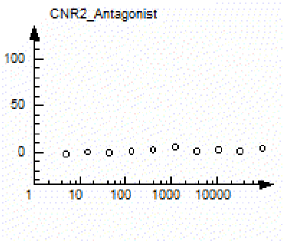

VBJ103
DRD1 Agonist
RC50 (uM) > 100

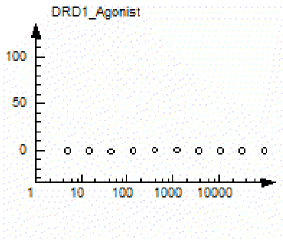

VBJ103
DRD1 Antagonist
RC50 (uM) > 100

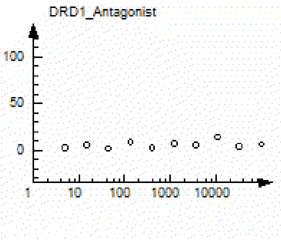

VBJ103
DRD2S Agonist
RC50 (uM) > 100

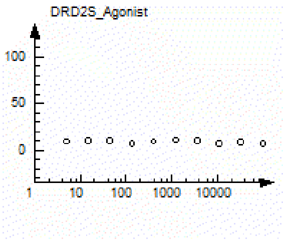

VBJ103
DRD2S Antagonist
RC50 (uM) > 100

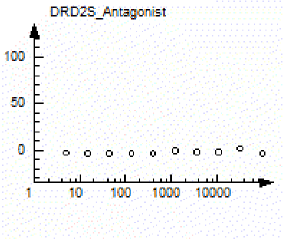

VBJ103
EDNRA Agonist
RC50 (uM) > 100

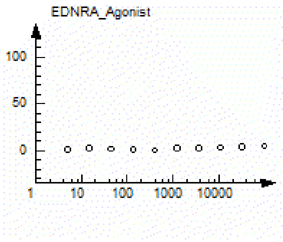

VBJ103
EDNRA Antagonist
RC50 (uM) > 100

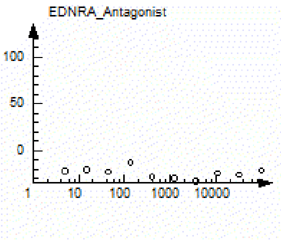

VBJ103
HRH1 Agonist
RC50 (uM) > 100

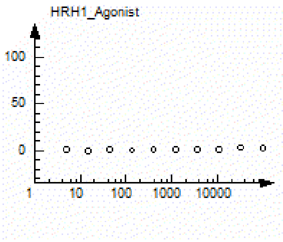

VBJ103
HRH1 Antagonist
RC50 (uM) > 100

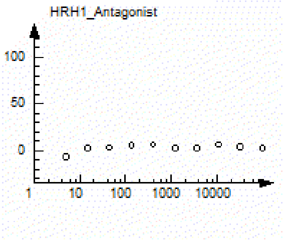

VBJ103
HRH2 Agonist
RC50 (uM) > 100

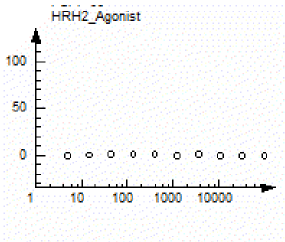

VBJ103
HRH2 Antagonist
RC50 (uM) > 100

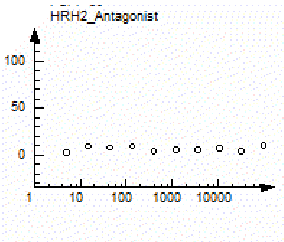

VBJ103
HTR1A Agonist
RC50 (uM) > 100

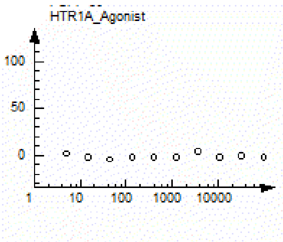

VBJ103
HTR1A Antagonist
RC50 (uM) > 100

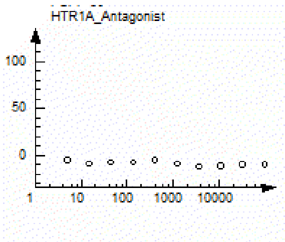

VBJ103
HTR1B Agonist
RC50 (uM) > 100

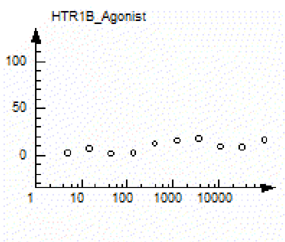

VBJ103
HTR1B Antagonist
RC50 (uM) > 100

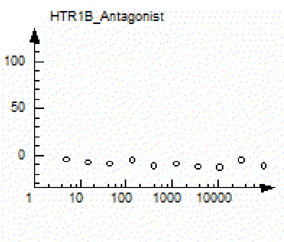

VBJ103
HTR2A Agonist
RC50 (uM) > 100

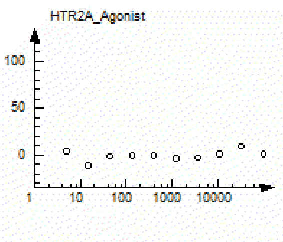

VBJ103
HTR2A Antagonist
RC50 (uM) > 100

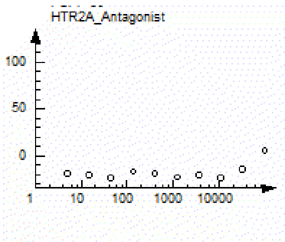

VBJ103
HTR2B Agonist
RC50 (uM) > 100

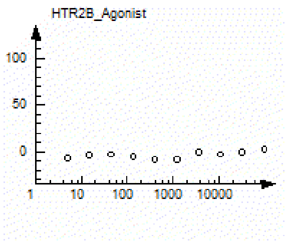

VBJ103
HTR2B Antagonist
RC50 (uM) > 100

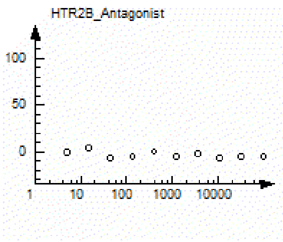

VBJ103
OPRD1 Agonist
RC50 (uM) > 100

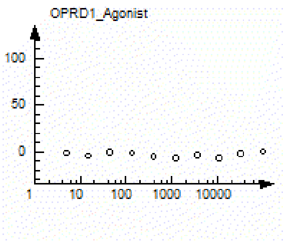

VBJ103
OPRD1 Antagonist
RC50 (uM) > 100

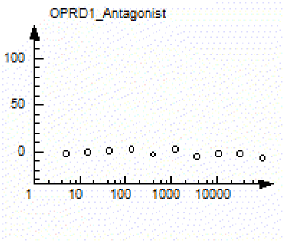

VBJ103
OPRK1 Agonist
RC50 (uM) > 100

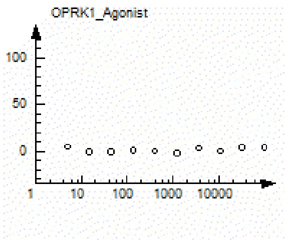

VBJ103
OPRK1 Antagonist
RC50 (uM) > 100

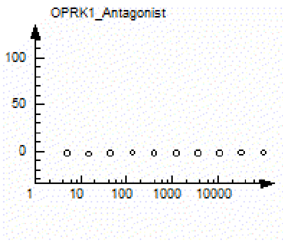

VBJ103
OPRM1 Agonist
RC50 (uM) > 100

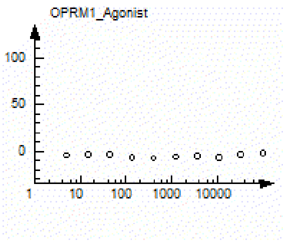

VBJ103
OPRM1 Antagonist
RC50 (uM) > 100

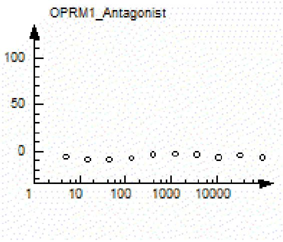

VBJ103
CAV1.2
RC50 (uM) > 100

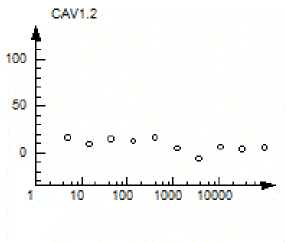

VBJ103
GABAA Antagonist
RC50 (uM) > 100

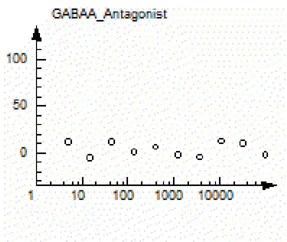

VBJ103
GABAA Agonist
RC50 (uM) > 100

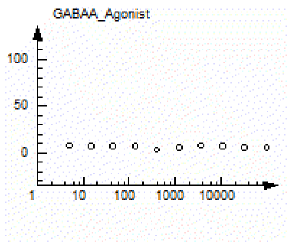

VBJ103
hERG
RC50 (uM) > 100

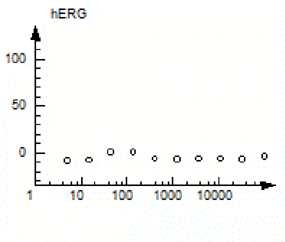

VBJ103
HTR3A Antagonist
RC50 (uM) > 100

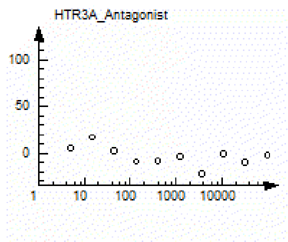

VBJ103
HTR3A Agonist
RC50 (uM) > 100

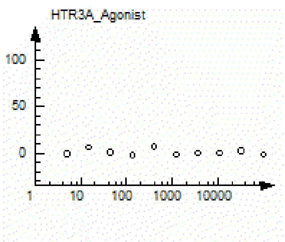

VBJ103
KvLQT1/minK Antagonist
RC50 (uM) > 100

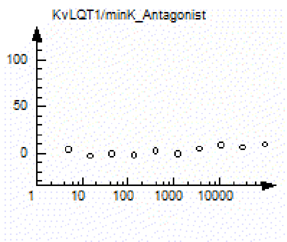

VBJ103
KvLQT1/minK Agonist
RC50 (uM) > 100

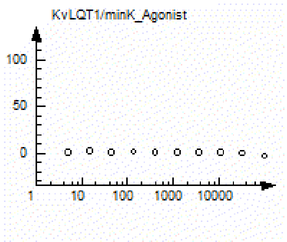

VBJ103
nAChR(a4/b2) Antagonist
RC50 (uM) > 100

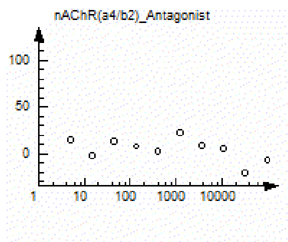

VBJ103
nAChR(a4/b2) Agonist
RC50 (uM) > 100

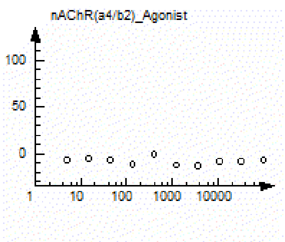

VBJ103
NAV1.5
RC50 (uM) > 100

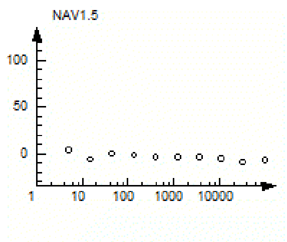

VBJ103
NMDAR 1A/2B Antagonist
RC50 (uM) > 100

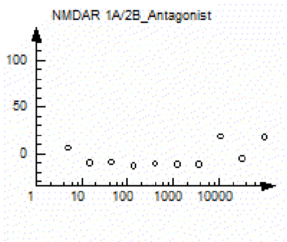

VBJ103
NMDAR 1A/2B Agonist
RC50 (uM) > 100

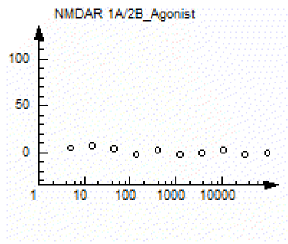

VBJ103
INSR
RC50 (uM) > 100

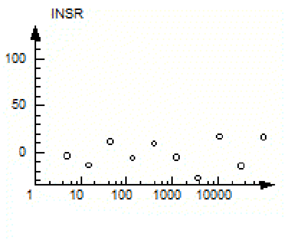

VBJ103
LCK
RC50 (uM) > 100

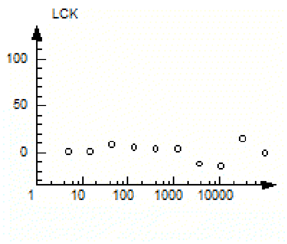

VBJ103
ROCK1
RC50 (uM) > 100

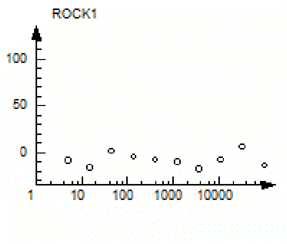

VBJ103
VEGFR2
RC50 (uM) > 100

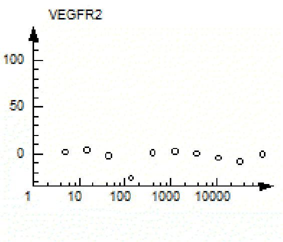

VBJ103
AR Agonist
RC50 (uM) > 100

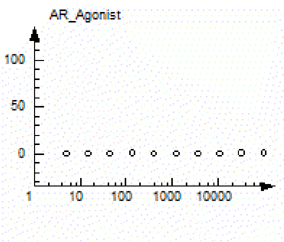

VBJ103
AR Antagonist
RC50 (uM) > 100

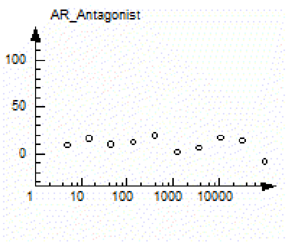

VBJ103
GR Agonist
RC50 (uM) > 100

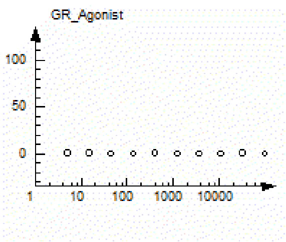

VBJ103
GR Antagonist
RC50 (uM) > 100

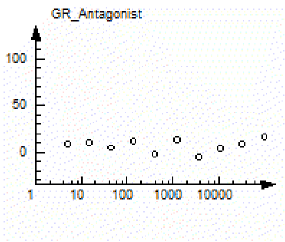

VBJ103
AChE
RC50 (uM) > 100

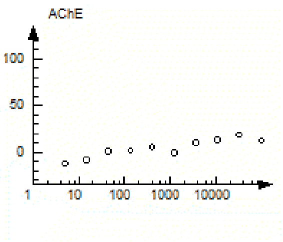

VBJ103
COX1
RC50 (uM) > 100

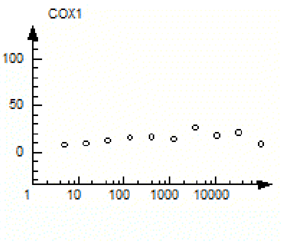

VBJ103
COX2
RC50 (uM) > 100

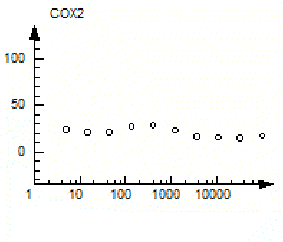

VBJ103
MAOA
RC50 (uM) > 100

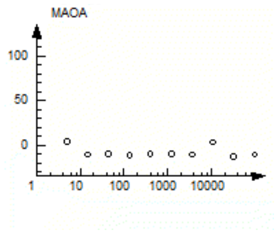

VBJ103
PDE3A
RC50 (uM) > 100

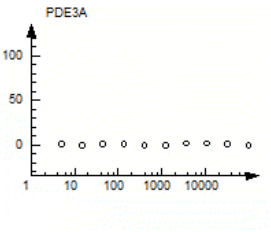

VBJ103
PDE4D2
RC50 (uM) > 100

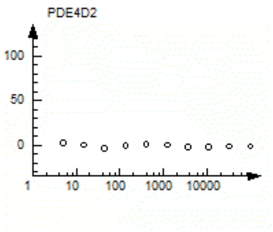

VBJ103
DAT
RC50 (uM) = 4.98494

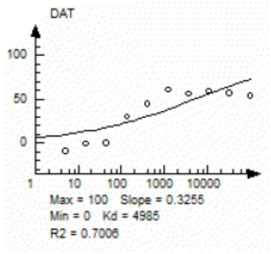

VBJ103
NET
RC50 (uM) > 100

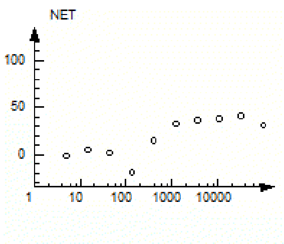

VBJ103
SERT
RC50 (uM) > 100

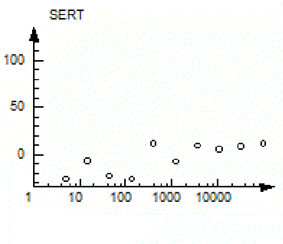

Figure S2. Concentration- response curves of VBJ103 across 78 assays tested in the SAFETYscan E/IC_{50} ELECT panel of human targets. Data shown are normalized to the maximal and minimal response observed in the presence of control ligand and vehicle respectively (y-axis) and is plotted against the corresponding compound concentration in nM in log10 scale (x-axis).

Target Class	Assay Name	Mode	Assay Target	Result Type	Value Prefix	RC50 (μM)	Hill	Curve Bottom	Curve Top	Max Response
GPCR	Calcium Flux	Agonist	ADORA2A	EC50	>	100				0
GPCR	Calcium Flux	Agonist	ADRA1A	EC50	>	100				13.66
GPCR	Calcium Flux	Agonist	AVPR1A	EC50	>	100				3.48
GPCR	Calcium Flux	Agonist	CCKAR	EC50	>	100				21.08
GPCR	Calcium Flux	Agonist	CHRM1	EC50	>	100				1.28
GPCR	Calcium Flux	Agonist	CHRM3	EC50	>	100				1.53
GPCR	Calcium Flux	Agonist	EDNRA	EC50	>	100				4.18
GPCR	Calcium Flux	Agonist	HRH1	EC50	>	100				2.64
GPCR	Calcium Flux	Agonist	HTR2A	EC50	>	100				8.46
GPCR	Calcium Flux	Agonist	HTR2B	EC50	>	100				1.35
GPCR	Calcium Flux	Antagonist	ADORA2A	IC50	>	100				5.22
GPCR	Calcium Flux	Antagonist	ADRA1A	IC50	>	100				0
GPCR	Calcium Flux	Antagonist	AVPR1A	IC50	>	100				0
GPCR	Calcium Flux	Antagonist	CCKAR	IC50	>	100				0
GPCR	Calcium Flux	Antagonist	CHRM1	IC50	>	100				0
GPCR	Calcium Flux	Antagonist	CHRM3	IC50	>	100				0.11
GPCR	Calcium Flux	Antagonist	EDNRA	IC50	>	100				0
GPCR	Calcium Flux	Antagonist	HRH1	IC50	>	100				3.34
GPCR	Calcium Flux	Antagonist	HTR2A	IC50	>	100				4.96
GPCR	Calcium Flux	Antagonist	HTR2B	IC50	>	100				0
GPCR	cAMP	Agonist	ADRA2A	EC50	>	100				2.72
GPCR	cAMP	Agonist	ADRB1	EC50	>	100				0
GPCR	cAMP	Agonist	ADRB2	EC50	>	100				0
GPCR	cAMP	Agonist	CHRM2	EC50	>	100				0
GPCR	cAMP	Agonist	CNR1	EC50	>	100				0
GPCR	cAMP	Agonist	CNR2	EC50	>	100				0

Table S9. Summary of Results for VBJ103 from the SAFETYscan E/IC_{50} ELECT panel.

Target Class	Assay Name	Mode	Assay Target	Result Type	Value Prefix	RC50 (μM)	Hill	Curve Bottom	Curve Top	Max Response
GPCR	cAMP	Agonist	DRD1	EC50	>	100				0
GPCR	cAMP	Agonist	DRD2S	EC50	>	100				7.65
GPCR	cAMP	Agonist	HRH2	EC50	>	100				0
GPCR	cAMP	Agonist	HTR1A	EC50	>	100				0
GPCR	cAMP	Agonist	HTR1B	EC50	>	100				16
GPCR	cAMP	Agonist	OPRD1	EC50	>	100				0
GPCR	cAMP	Agonist	OPRK1	EC50	>	100				3.4
GPCR	cAMP	Agonist	OPRM1	EC50	>	100				0
GPCR	cAMP	Antagonist	ADRA2A	IC50	>	100				0
GPCR	cAMP	Antagonist	ADRB1	IC50	>	100				12.61
GPCR	cAMP	Antagonist	ADRB2	IC50	>	100				21.3
GPCR	cAMP	Antagonist	CHRM2	IC50	>	100				6.91
GPCR	cAMP	Antagonist	CNR1	IC50	>	100				15.37
GPCR	cAMP	Antagonist	CNR2	IC50	>	100				2.82
GPCR	cAMP	Antagonist	DRD1	IC50	>	100				5.79
GPCR	cAMP	Antagonist	DRD2S	IC50	>	100				0.91
GPCR	cAMP	Antagonist	HRH2	IC50	>	100				9.41
GPCR	cAMP	Antagonist	HTR1A	IC50	>	100				0
GPCR	cAMP	Antagonist	HTR1B	IC50	>	100				0
GPCR	cAMP	Antagonist	OPRD1	IC50	>	100				0
GPCR	cAMP	Antagonist	OPRK1	IC50	>	100				0
GPCR	cAMP	Antagonist	OPRM1	IC50	>	100				0
Ion Channel	Ion Channel	Blocker	CAV1.2	IC50	>	100				5.11
Ion Channel	Ion Channel	Blocker	GABAA	IC50	>	100				9.58
Ion Channel	Ion Channel	Blocker	hERG	IC50	>	100				0
Ion Channel	Ion Channel	Blocker	HTR3A	IC50	>	100				0

Table S9 (contd).

Target Class	Assay Name	Mode	Assay Target	Result Type	Value Prefix	RC50 (µM)	Hill	Curve Bottom	Curve Top	Max Response
Ion Channel	Ion Channel	Blocker	KvLQT1/minK	IC50	>	100				8.47
Ion Channel	Ion Channel	Blocker	nAChR(a4/b2)	IC50	>	100				0
Ion Channel	Ion Channel	Blocker	NAV1.5	IC50	>	100				0
Ion Channel	Ion Channel	Blocker	NMDAR (1A/2B)	IC50	>	100				16.95
Ion Channel	Ion Channel	Opener	GABAA	EC50	>	100				4.94
Ion Channel	Ion Channel	Opener	HTR3A	EC50	>	100				1.63
Ion Channel	Ion Channel	Opener	KvLQT1/minK	EC50	>	100				0
Ion Channel	Ion Channel	Opener	nAChR(a4/b2)	EC50	>	100				0
Ion Channel	Ion Channel	Opener	NMDAR (1A/2B)	EC50	>	100				0
Kinases	Binding	Inhibitor	INSR	IC50	>	100				15.13
Kinases	Binding	Inhibitor	LCK	IC50	>	100				14.25
Kinases	Binding	Inhibitor	ROCK1	IC50	>	100				5.38
Kinases	Binding	Inhibitor	VEGFR2	IC50	>	100				0
NHR	NHR Nuclear Translocation	Agonist	AR	EC50	>	100				0.15
NHR	NHR Nuclear Translocation	Antagonist	AR	IC50	>	100				13.4
NHR	NHR Protein Interaction	Agonist	GR	EC50	>	100				0.04
NHR	NHR Protein Interaction	Antagonist	GR	IC50	>	100				16.05
Non-Kinase Enzymes	Enzymatic	Inhibitor	AChE	IC50	>	100				17.64
Non-Kinase Enzymes	Enzymatic	Inhibitor	COX1	IC50	>	100				20.07
Non-Kinase Enzymes	Enzymatic	Inhibitor	COX2	IC50	>	100				16.31
Non-Kinase Enzymes	Enzymatic	Inhibitor	MAOA	IC50	>	100				0
Non-Kinase Enzymes	Enzymatic	Inhibitor	PDE3A	IC50	>	100				0
Non-Kinase Enzymes	Enzymatic	Inhibitor	PDE4D2	IC50	>	100				0
Transporter	Transporter	Blocker	DAT	IC50	=	4.98494	0.33	0	100	56.27
Transporter	Transporter	Blocker	NET	IC50	>	100				40.04
Transporter	Transporter	Blocker	SERT	IC50	>	100				11.2

Table S9 (contd).

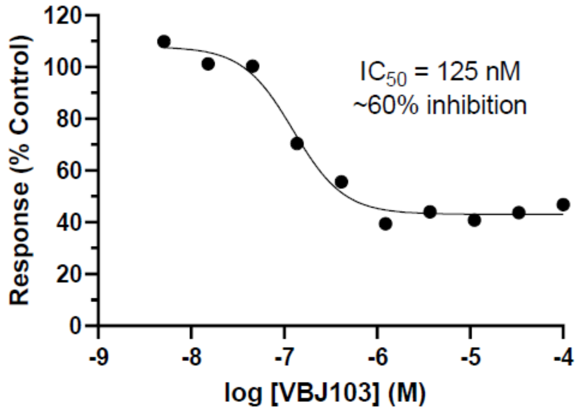

Figure S3. Antagonist activity of VBJ103 against the dopamine transporter (DAT). Percent inhibition is in relation to the control GBR 12909 (IC$_{50}$: 0.00547 μM). Data represent a single experiment.

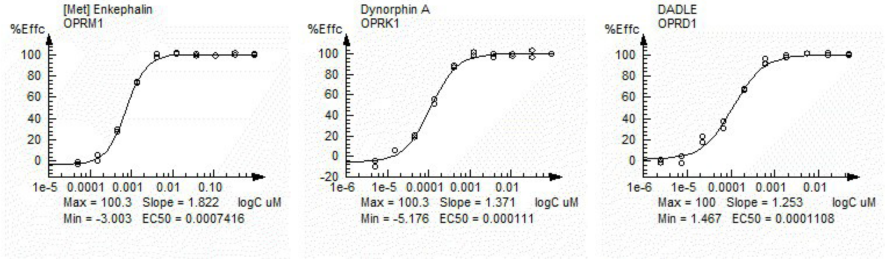

Figure S4. Control dose response curves for the Hit Hunter® cAMP assay at mu-, kappa- and delta-opioid receptors. Data shown was normalized to the maximal and minimal response observed in the presence of control compound and vehicle respectively. For Gi cAMP assays, the following forskolin concentration was used: OPRM1 cAMP: 20 μM Forskolin; OPRK1 cAMP: 15 μM Forskolin; OPRD1 cAMP: 20 μM Forskolin.

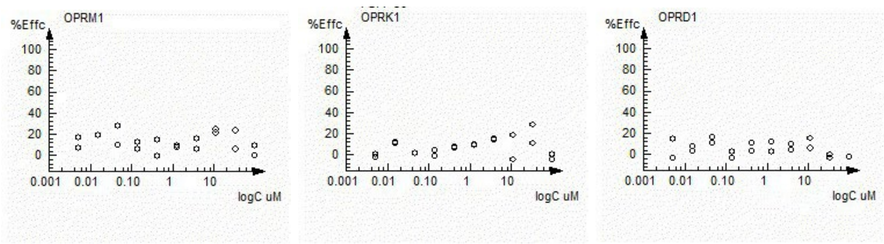

Figure S5. VBJ103 activity in the Hit Hunter® cAMP assay at mu-, kappa- and delta-opioid receptors tested in PAM mode. For PAM assays, data was normalized to the

maximal and minimal response observed in the presence of EC_{20} ligand and vehicle. The following EC_{20} concentrations were used: OPRM1 cAMP: 0.00025 μM [Met] Enkephalin; OPRK1 cAMP: 0.00003 μM Dynorphin A; OPRD1 cAMP: 0.00002 μM DADLE. For Gi cAMP assays, the following forskolin concentration was used: OPRM1 cAMP: 20 μM Forskolin; OPRK1 cAMP: 15 μM Forskolin; OPRD1 cAMP: 20 μM Forskolin.

Compound Name	Project ID	Assay Name	Assay Format	Assay Target	Result Type	RC50	Unit	Hill	Curve Bottom	Curve Top	Max Response
DADLE	US034-0013850-O	cAMP	Agonist	OPRD1	EC50	0.0001108358	uM	1.2527	1.4666	100	100.31
Dynorphin A	US034-0013850-O	cAMP	Agonist	OPRK1	EC50	0.0001110018	uM	1.3714	-5.1757	100.32	100
[Met] Enkephalin	US034-0013850-O	cAMP	Agonist	OPRM1	EC50	0.0007416345	uM	1.8218	-3.0033	100.31	100.33
VBJ103	US034-0013850-O	cAMP	Positive Allosteric Modulator	OPRD1	EC50	>100	uM				0
VBJ103	US034-0013850-O	cAMP	Positive Allosteric Modulator	OPRK1	EC50	>100	uM				19.7
VBJ103	US034-0013850-O	cAMP	Positive Allosteric Modulator	OPRM1	EC50	>100	uM				14.54

Table S10. Summary of Results from the Hit Hunter® cAMP assays at mu-, kappa- and delta-opioid receptors.

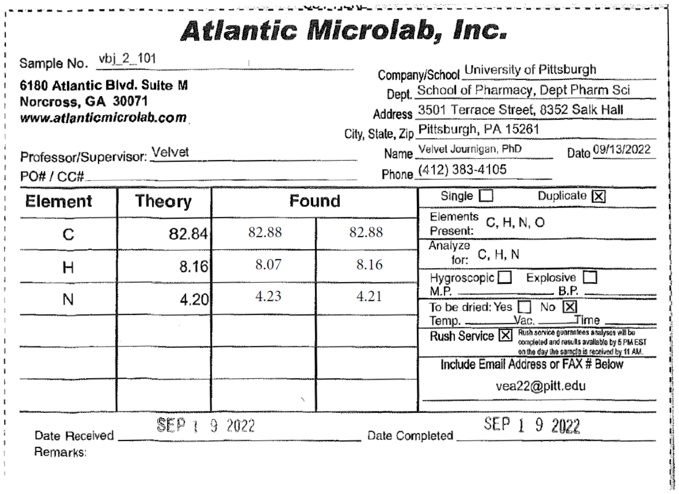

Atlantic Microlab, Inc.

Sample No. vbj_2_101

6180 Atlantic Blvd. Suite M
Norcross, GA 30071
www.atlanticmicrolab.com

Company/School University of Pittsburgh
Dept. School of Pharmacy, Dept Pharm Sci
Address 3501 Terrace Street, 8352 Salk Hall
City, State, Zip Pittsburgh, PA 15261
Name Velvet Journigan, PhD Date 09/13/2022
Phone (412) 383-4105

Professor/Supervisor: Velvet
PO# / CC#

Element	Theory	Found	
C	82.84	82.88	82.88
H	8.16	8.07	8.16
N	4.20	4.23	4.21

Single ☐ Duplicate ☒
Elements Present: C, H, N, O
Analyze for: C, H, N
Hygroscopic ☐ Explosive ☐
M.P. ____ B.P. ____
To be dried: Yes ☐ No ☒
Temp. ____ Vac. ____ Time ____
Rush Service ☒ Rush service guarantees analyses will be completed and results available by 5 PM EST on the day the sample is received by 11 AM.
Include Email Address or FAX # Below
vea22@pitt.edu

Date Received SEP 1 9 2022 Date Completed SEP 1 9 2022
Remarks:

Figure S6. Combustion (elemental) analysis results of VBJ103

Note, vbj_2_101 is the internal code numbering for the current batch of VBJ103 tested in vivo

	logD (pH 7.4)	Mouse Liver microsomes, $T_{1/2}$ (min) [a]
**VBJ103**	3.21	30
**testosterone**	3.09	3.7

[a] Journigan et al, 2020

Table S11. Solubility and metabolic stability of VBJ103

Time (h)	VBJ103 Levels							
	Male Rats (µM)				Female Rats (µM)			
	Animal 23	Animal 24	Animal 25		Animal 26	Animal 27	Animal 28	
1	0.053	0.047	0.056		0.040	0.039	0.051	
	Animal 9	Animal 10	Animal 11	Animal 12	Animal 13	Animal 14	Animal 15	Animal 16
24	0.0079	0.0039	0.0049	0.0069	0.0146	0.0119	0.0071	0.0081

Table S12. Brain Levels of VBJ103 (100 mg/kg) in Male and Female SD Rats Following SC administration.

Rat #	Sex	Body Weight (g)	Brain Weight (mg)	Dosing Volume (mL)	Homogenate Volume (µL)
9	M	316	1899.3	1.58	9497
10	M	318	1708.9	1.59	8545
11	M	342	1839.6	1.71	9198
12	M	322	1771.5	1.61	8858
13	F	276	1833.8	1.38	9169
14	F	252	1707.2	1.26	8536
15	F	255	1757.2	1.28	8786
16	F	244	1776.4	1.22	8882
23	M	366	1865.9	1.83	9330
24	M	360	1778.4	1.8	8892
25	M	353	1806.2	1.77	9031
26	F	222	1747.1	1.11	8736
27	F	244	1849.2	1.22	9246
28	F	242	1799.1	1.21	8996

Table S13. VBJ103 (100 mg/kg) brain weights. Initially, 4x the volume of homogenization solution was added based on brain weight. The actual total volume of the homogenate includes the solution volume and brain volume. Ultimately 5x the brain mass provided the total homogenate volume listed in µL.
